# Recruitment of CXCR4^+^ type 1 innate lymphoid cells distinguishes sarcoidosis from other skin granulomatous diseases

**DOI:** 10.1172/JCI178711

**Published:** 2024-09-03

**Authors:** Satish Sati, Jianhe Huang, Anna E. Kersh, Parker Jones, Olivia Ahart, Christina Murphy, Stephen M. Prouty, Matthew L. Hedberg, Vaibhav Jain, Simon G. Gregory, Denis H. Leung, John T. Seykora, Misha Rosenbach, Thomas H. Leung

**Affiliations:** 1 Department of Dermatology, University of Pennsylvania School of Medicine, Philadelphia, Pennsylvania, USA.; 2 Duke Molecular Physiology Institute, Durham, North Carolina, USA.; 3 Singapore Management University, Singapore.; 4 Corporal Michael Crescenz Veterans Affairs Medical Center, Philadelphia, Pennsylvania, USA.

**Keywords:** Dermatology, Immunology, Cellular immune response, Innate immunity

## Abstract

Sarcoidosis is a multiorgan granulomatous disease that lacks diagnostic biomarkers and targeted treatments. Using blood and skin from patients with sarcoid and non-sarcoid skin granulomas, we discovered that skin granulomas from different diseases exhibit unique immune cell recruitment and molecular signatures. Sarcoid skin granulomas were specifically enriched for type 1 innate lymphoid cells (ILC1s) and B cells and exhibited molecular programs associated with formation of mature tertiary lymphoid structures (TLSs), including increased CXCL12/CXCR4 signaling. Lung sarcoidosis granulomas also displayed similar immune cell recruitment. Thus, granuloma formation was not a generic molecular response. In addition to tissue-specific effects, patients with sarcoidosis exhibited an 8-fold increase in circulating ILC1s, which correlated with treatment status. Multiple immune cell types induced CXCL12/CXCR4 signaling in sarcoidosis, including Th1 T cells, macrophages, and ILCs. Mechanistically, CXCR4 inhibition reduced sarcoidosis-activated immune cell migration, and targeting CXCR4 or total ILCs attenuated granuloma formation in a noninfectious mouse model. Taken together, our results show that ILC1s are a tissue and circulating biomarker that distinguishes sarcoidosis from other skin granulomatous diseases. Repurposing existing CXCR4 inhibitors may offer a new targeted treatment for this devastating disease.

## Abstract 2

Type 1 innate lymphoid cells serve as a diagnostic biomarker for sarcoidosis, and the CXCR4 inhibitor plerixafor, an FDA-approved drug, may provide a targeted treatment.

## Graphical abstract

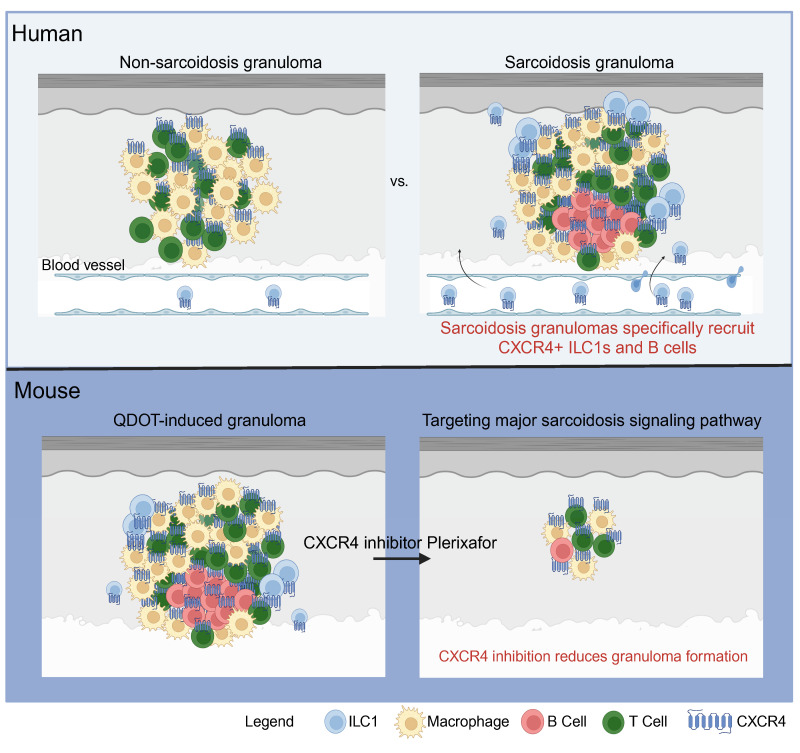

## Introduction

Sarcoidosis remains a debilitating, and sometimes fatal, multiorgan inflammatory granulomatous disease that predominately affects the lung and skin. Patients struggle with fatigue, difficulty breathing, and pain in their joints, eyes, and skin. Despite advances in modern medicine, sarcoidosis diagnosis requires active exclusion of other diseases, delaying care and initiation of treatment (1). Monitoring disease progression also remains difficult, and current treatments, such as steroids and other immunosuppressive agents, have serious well-described side-effects (2, 3). Thus, biomarkers for sarcoidosis diagnosis and disease activity, as well as targeted treatments, remain major unmet clinical needs.

Granulomas are defined clusters of immune cells deposited in organs, and sarcoid granuloma formation is thought to begin with presentation of an unknown antigen by macrophages and/or dendritic cells (DCs) to CD4^+^ T helper cells (4). Prior work demonstrated that the periphery of sarcoid granulomas contains other types of immune cells, including CD8^+^ T cells, type 17.1 helper T (Th17.1) cells, and B cells. The role of these cell types in sarcoid granuloma formation is not well understood (5).

Innate lymphoid cells (ILCs) are the innate counterparts of T cells and lack the adaptive antigen receptor machinery. There are 4 subtypes of ILCs (ILC1, ILC2, ILC3, and natural killer [NK] cells), and each one functionally mirrors the cytokine production of a different T cell subtype (6). ILCs localize into barrier tissues during development and may also be recruited from circulating progenitor cells, especially during infection (7–11). ILC2s and ILC3s regulate granuloma formation during microbial infections (12–14). However, the role of ILCs in sarcoid granuloma formation, for which an infectious trigger has not been established, and other noninfectious granulomatous diseases remains unknown.

Tertiary lymphoid structures (TLSs) are organized aggregates of immune cells that are different from granulomas and resemble secondary lymphoid organs in nonlymphoid tissues (15). They arise in the context of chronic inflammation, including cancer, infections, and granulomatous diseases (such as Crohn disease). TLSs undergo maturation through 3 different stages. Lymphoid aggregates are the least organized stage, with the absence of CD21^+^ follicular DCs and lack of segregated T and B cell zones. Non–germinal center TLSs contain CD21^+^ follicular DCs, but lack germinal center–like B cells. Finally, mature TLSs recruit both CD21^+^ follicular DCs and germinal center–like B cells, the interactions of which are tightly regulated by cytokines, including CXCL13 and CCL19 (16, 17). While sarcoid granulomas do not typically develop germinal centers, the role of B cells and TLSs in sarcoidosis and other skin granulomatous diseases remains undefined.

In addition to sarcoidosis, skin granulomas may also develop in the setting of other inflammatory disorders. We performed high-resolution analysis of skin and blood samples from patients with non-sarcoid and sarcoid skin granulomas to assess disease-specific differences in granuloma formation. We show that ILC1s specifically traffic to sarcoid granulomas, and sarcoid granulomas uniquely exhibit molecular signaling programs associated with mature TLSs, including CXCL12/CXCR4 activation.

## Results

### Immune cell landscape in sarcoid and non-sarcoid skin granulomas.

**Figure 1:**
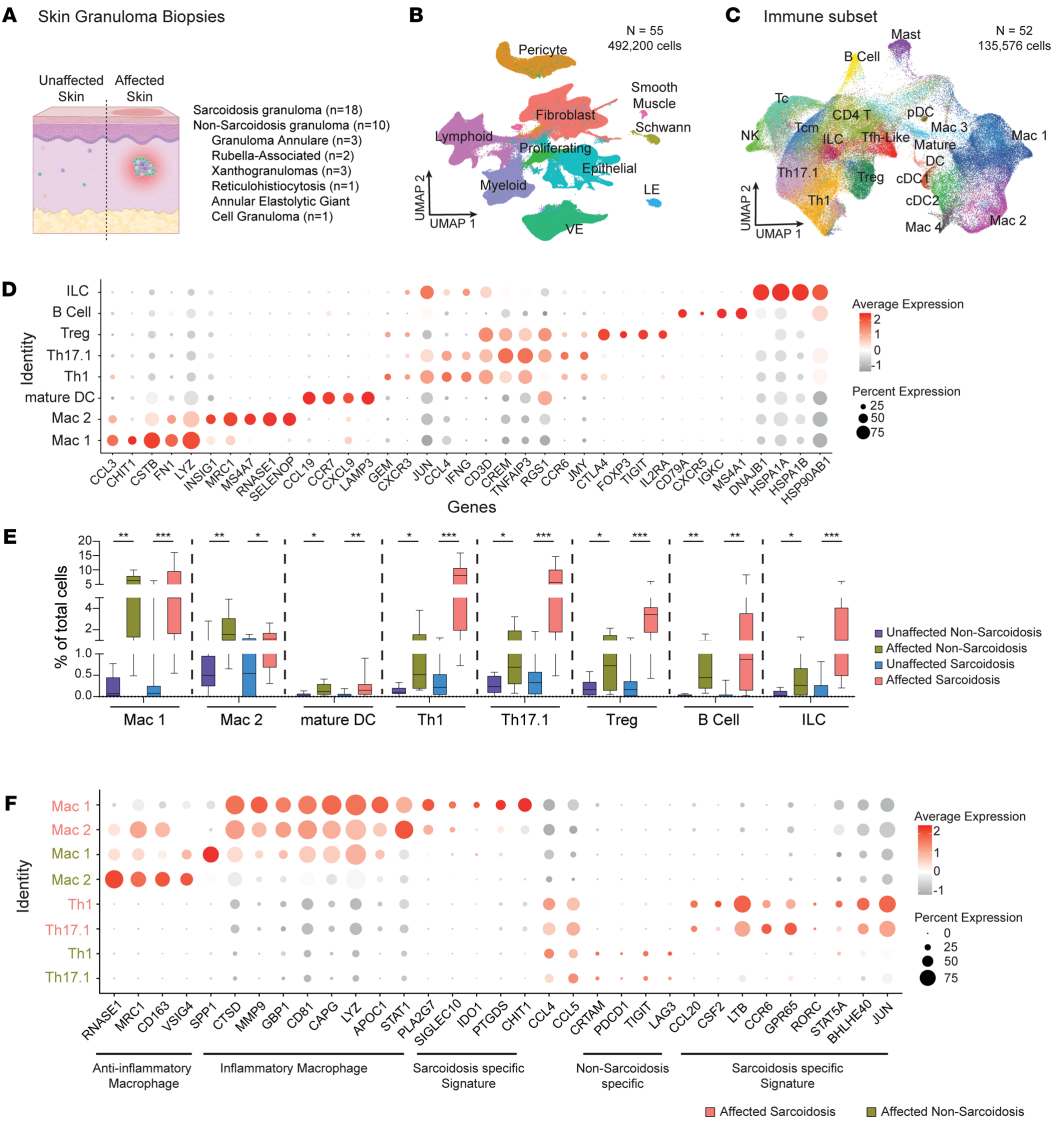
Immune cell landscape in sarcoidosis and non-sarcoidosis skin granulomas. (**A**) Overview of sample collection. (**B**) Identification of cell clusters from patients with sarcoidosis (*n* = 18 for affected and *n* = 18 unaffected skin) and non-sarcoidosis skin granuloma patients (*n* = 10 for affected and *n* = 9 unaffected skin). VE, vascular endothelium; LE, lymphatic endothelium. (**C**) UMAP depicting subclustering of immune cells. pDC, plasmacytoid DC; cDC, conventional DC. (**D**) Marker genes defining immune subsets. Dot size reflects percentage cells expressing the gene, and color illustrates level of gene expression. (**E**) Box-and-whisker plot shows relative contribution of immune cells as percentage of total cells. In the box-and-whisker plot, the box extends from the 25th to 75th percentile. The line in the middle of the box represents the median, and the whiskers represent the minimum and maximum. All data points are covered, no outlying values. Statistical significance was calculated using a 2-tailed Student’s *t* test. (**F**) Dot plot depicting gene activation in different immune clusters. Dot size reflects percentage cells expressing the gene, and color illustrates level of gene expression. Data depicted as mean ± SEM. *P < 0.05; **P < 0.01; ***P < 0.001.

We collected 4-mm skin biopsies (affected and unaffected skin) from 18 patients with sarcoid and 10 with non-sarcoid skin granulomas with clinically active and histologically validated skin disease (Figure 1A and Supplemental Figure 1A; supplemental material available online with this article; https://doi.org/10.1172/JCI178711DS1). Unaffected skin biopsies from the same donor served as control samples. Non-sarcoidosis patients were diagnosed with granuloma annulare, xanthogranuloma, multicentric reticulohistiocytosis, annular elastolytic giant cell granuloma, or rubella virus–associated skin granulomatous dermatitis (Supplemental Table 1). In our sarcoidosis cohort, we had 9 male and 9 female patients, with a mean age of 54 years; 56% of the patients were Black/African American. Most patients exhibited disease in multiple organs (Supplemental Table 1). In our non-sarcoidosis cohort, we had 3 male and 7 female patients, with a mean age of 68 years. We generated 492,200 high-quality single-cell RNA-sequencing (scRNA-seq) profiles (Supplemental Table 2). Unsupervised clustering of scRNA-seq profiles identified 59 cell clusters, which were annotated to 10 cell types based on marker gene identification, lineage marker genes, and mapping to single-cell databases (Figure 1B, Supplemental Figure 1, B–E, and Supplemental Table 3). The identified cell types were shared among affected and unaffected samples (Supplemental Figure 1, B–F).

We tracked the immune cell contributions to non-sarcoid and sarcoid granulomas. We subclustered the immune cell populations and identified 19 different subtypes (Figure 1C, marker genes for each subtype are listed in Figure 1D, Supplemental Figure 2, A–D, and Supplemental Table 3). Within the myeloid lineage, we identified 4 distinct subsets of macrophages in all samples, with Mac 1 and Mac 2 being the dominant populations. Mac 1 adopted an inflammatory phenotype, including expression of inflammatory marker genes: *CCL3*, *CSTB*, *FN1*, and *LYZ*. Mac 2 exhibited an antiinflammatory phenotype, including expression of antiinflammatory marker genes: *CD163*, *MRC1*, *MS4A7*, and *SELENOP*. Both sarcoidosis- and non–sarcoidosis-affected skin recruited more myeloid cells compared with unaffected skin (Figure 1E). However, their activation states differed. Mac 1 and Mac 2 in non-sarcoid granulomas maintained inflammatory and antiinflammatory activation states, respectively (Figure 1F, green rows). In contrast, both Mac 1 and Mac 2 in sarcoid granulomas adopted an inflammatory state with sarcoidosis-specific gene activation, including *CHIT1*, *PTGDS*, and *PLA2G7*. PLA2G7-expressing macrophages have been shown to be highly immunosuppressive and impede T cell activation (18). Taken together, the results show that macrophages are recruited to similar levels in sarcoidosis and non-sarcoidosis skin granulomatous diseases, but exhibit different activation states. To be comprehensive, we also identified a population of mature DCs expressing *LAMP3* (Figure 1, D and E).

Within the lymphoid lineage, we identified 6 subtypes of T cells, B cells, and NK cells (Figure 1D and Supplemental Figure 2, C and D). The T subtypes were CD8^+^ cytotoxic (Tc) cells, central memory (Tcm) cells, follicular helper-like (Tfh-like) cells, CD4^+^ Th1 cells, Th17.1 cells, and regulatory (Treg) cells (Figure 1D and Supplemental Figure 2, C and D). Within sarcoidosis-affected skin, Th1 and Th17.1 T cell populations were the dominant subtypes; Th17.1 cells produced *IFNG*, but not *IL17A* (Supplemental Figure 4A) (19, 20). Overall, sarcoidosis-affected skin recruited more lymphoid cells across all subtypes compared with non–sarcoidosis-affected skin (Figure 1E and Supplemental Figure 3). Th1 and Th17.1 cells in sarcoidosis-affected skin induced NF-κB–associated inflammatory genes, including *LTB*, *TNF*, *CCR6*, and *IL6*. Similar to myeloid cells, sarcoidosis-activated lymphoid cells adopted a more inflammatory phenotype compared with non–sarcoidosis-activated lymphoid cells (Figure 1F). Surprisingly, lymphoid cells in non–sarcoidosis-affected skin specifically induced immune checkpoint genes, including *TIGIT*, *LAG3*, and *PDCD1*. *LAG3* and *PDCD1* are well known for negatively regulating T cell expansion and their effector functions such as cytokine secretion (21, 22). Thus, the well-known paradoxical immune response in sarcoidosis, characterized by both T cell anergy and T cell expansion, can potentially be explained by the involvement of immunosuppressive macrophages coupled with the lack of immune checkpoint gene induction in T cells. Finally, sarcoidosis-affected skin was enriched in B cells that expressed *CDA* and *BCL6*, 2 genes typically induced during late-stage changes in germinal center differentiation and isotype switching (Supplemental Figure 4B) (23, 24).

### Sarcoid granulomas specifically recruit ILC1s.

Next, we wanted to take advantage of our unbiased approach and identify sarcoidosis-specific immune cell populations. Within affected sarcoid skin, we identified a population matching ILCs by using previously identified marker genes (*HSPA1A*, *HSPA1B*, *IFNG*, *TNF*, *CXCR4*, and *TNFSF11*) (Figure 1D, Supplemental Figure 2C, and Supplemental Figure 3) (25, 26). Differential gene expression analysis also confirmed the absence of T cell receptor (TCR) genes in this ILC cluster (*TRA*, *CD3E*, and *CD247*) (Supplemental Tables 3–5). 3D uniform manifold approximation and projection (UMAP) and *t*-distributed stochastic neighbor embedding (tSNE) clustering further confirmed that this population was distinct from other T cell populations (Supplemental Figure 4, C and D). Affected sarcoid skin contained on average approximately 5-fold more ILCs compared with non–sarcoidosis-affected skin (Figure 1E).

**Figure 2:**
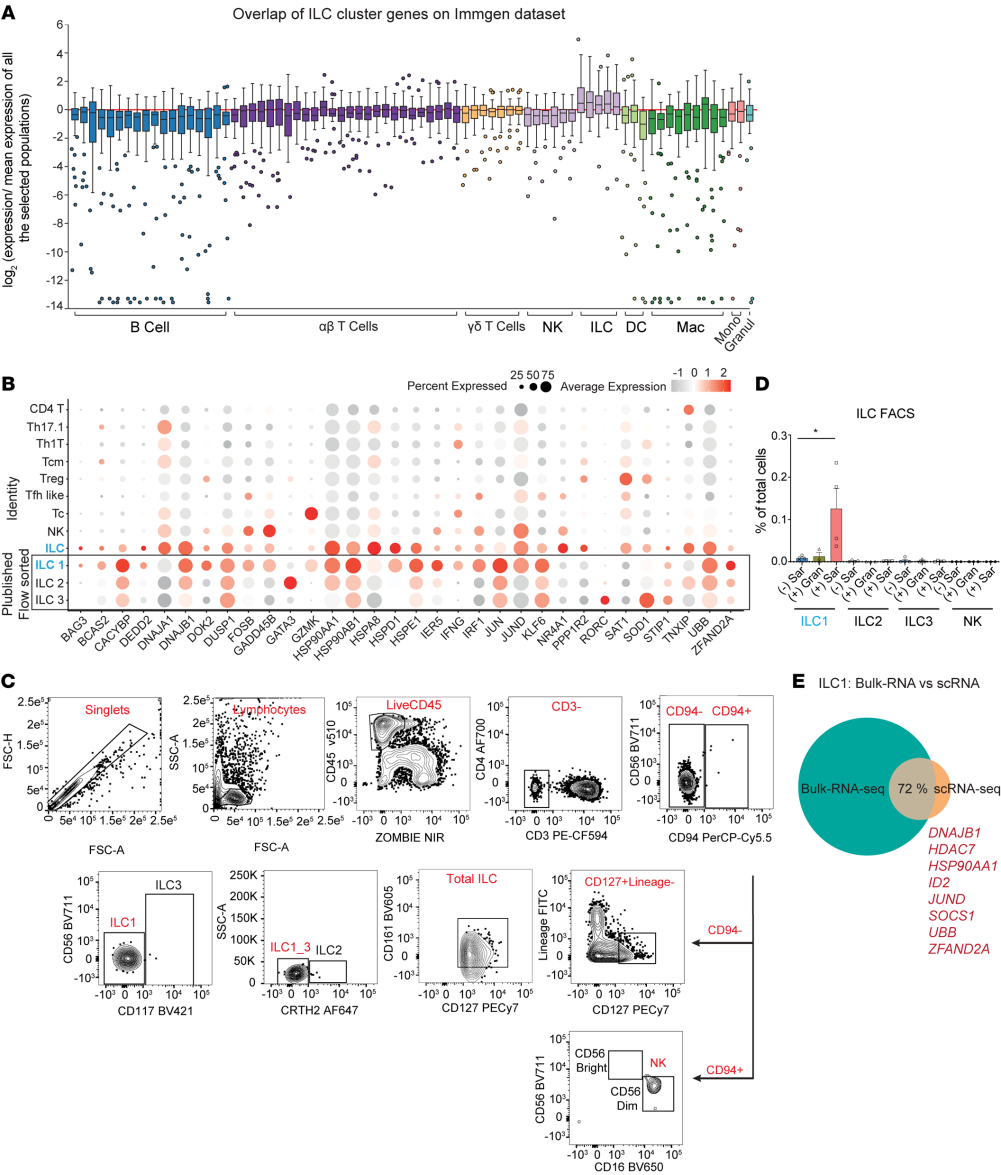
Group 1 ILCs are specifically recruited to sarcoid granulomas. (**A**) Gene expression profile of single-cell-identified ILC cluster best matched the ILC definition in the Immunological Genome Project (ImmGen) database. (**B**) Dot plot comparing gene expression profiles of sarcoidosis immune cells and ILCs to published purified ILC subpopulations. Sarcoidosis ILCs most closely matched ILC1. Dot size reflects percentage cells expressing the gene, and color illustrates level of gene expression. (**C**) Flow cytometry gating scheme for human ILCs, including NK cells. (**D**) Flow cytometry analysis of ILC subtypes as percentage of total sorted cells in affected and unaffected sarcoidosis and non-sarcoidosis granuloma skin (*n* = 3). One-way ANOVA revealed statistical significance for ILC1. *P < 0.05. (**E**) Comparison of gene expression from flow cytometry–purified sarcoidosis skin ILC1s (*n* = 3) to sarcoidosis ILCs identified in our scRNA-seq data sets. Eight out of top 10 genes matched and are listed.

We confirmed the identity of this ILC population using 4 additional methods. (a) Due to lack of a human skin immune cell reference atlas, we overlaid the gene expression profiles from our identified ILC subcluster with the mouse Immunological Genome Project (ImmGen) database (https://www.immgen.org/ Accessed April 30, 2023.), and this expression pattern matched their ILC profile (Figure 2A). (b) Given the mouse-human comparison, we also compared our gene expression profiles against published human data sets of flow cytometry–purified ILC subtypes (27). Our sarcoidosis ILCs most closely matched the ILC1 gene profile (Figure 2B). (c) We adapted a published flow cytometry gating strategy for all individual ILC subtypes, including ILC1, ILC2, ILC3, and NK cells (12, 28, 29) (Figure 2C). We performed flow cytometry on affected and unaffected skin from 4 patients with sarcoid and 4 with non-sarcoid skin granulomas. Affected sarcoid skin contained approximately 10-fold more ILCs, specifically ILC1s, compared with non–sarcoidosis-affected skin (Figure 2D). Non–sarcoidosis-affected skin did not display any enrichment for ILCs. (d) Finally, we used the same flow cytometry gating scheme to purify ILC1s from affected sarcoid skin and performed bulk RNA-seq (Supplemental Figure 4E). Using this data set as a reference, 72% of the top 100 genes within the ILC population identified in our scRNA-seq data set overlapped, including 8 of the top 10 expressed genes (Figure 2E). Taken together, our results show that sarcoidosis-affected skin specifically recruits more ILC1s.

Two other groups have generated scRNA-seq data sets from sarcoidosis-affected skin, and we found similar enrichment of ILCs in their data sets (Supplemental Figure 4, F–H) (30, 31). ILCs have been previously implicated in other inflammatory skin diseases, including psoriasis and atopic dermatitis. We compared ILCs from our sarcoidosis and non-sarcoidosis samples with ILCs identified from 6 other skin inflammatory conditions (32). ILCs from sarcoidosis-affected skin formed a distinct cluster, underscoring their unique nature and distinguishing them from ILCs in other skin inflammatory conditions (Supplemental Figure 5).

In summary, sarcoid and non-sarcoid skin granulomas exhibit disease-specific immune cell recruitment. Non-sarcoid granulomas displayed a macrophage-dominant response, and sarcoid granulomas exhibited a more complex immune response with Th1 cells, B cells, and ILC1s.

### Spatial organization of sarcoid and non-sarcoid granulomas.

We next assessed the spatial organization of B cells and ILCs within sarcoid skin granulomas using spatial transcriptomics and immunohistology. Visium spatial transcriptomics (10× Genomics) quantifies RNA transcripts within a 55-μm spot size, and sarcoid granulomas are often 200–400 μm in size, which enables determining whether different immune cell populations are located at tissue granulomas. Spatial transcriptomes generated from affected and unaffected skin from 2 patients with sarcoidosis and 1 non-sarcoidosis patient yielded 9,272 spots at an average depth of approximately 1,159 spots per sample and approximately 1,127 genes/spot (Supplemental Table 2). These patients were part of our skin scRNA-seq cohort. We performed UMAP dimensionality reduction and unsupervised clustering to identify 10 cell clusters. These clusters were annotated to cell types based on marker gene identification, lineage marker genes, and mapping of spot gene signature to single-cell databases (Supplemental Figure 6, A and B, and Supplemental Table 3).

**Figure 3:**
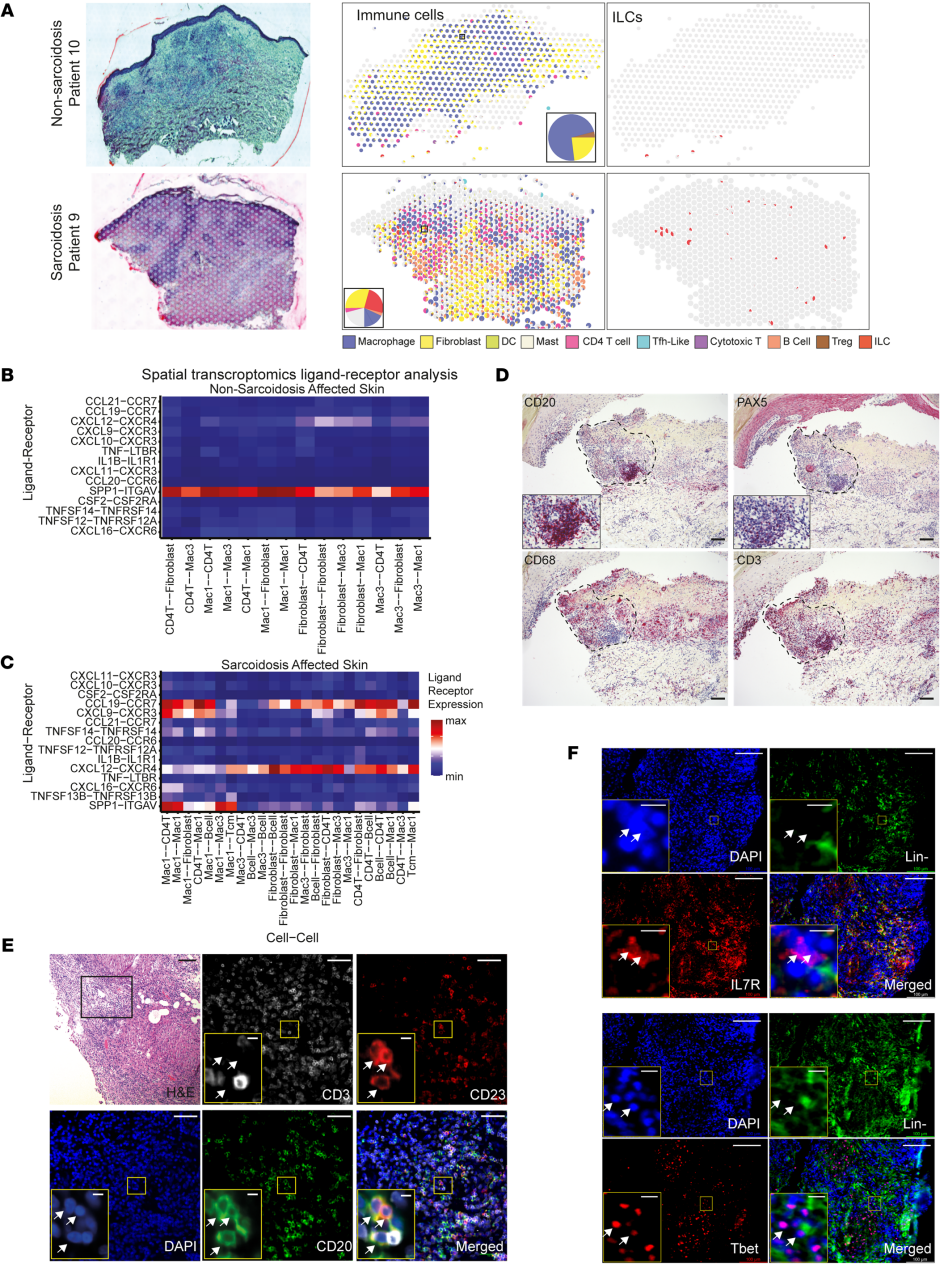
Sarcoid granulomas contain ILC1s and B cell aggregates. (**A**) Deconvoluted cell type identification from spatial transcriptomics of patients with sarcoidosis (*n* = 2) and non-sarcoidosis granuloma (*n* = 1) patients. Each spot is represented as a pie chart displaying relative cell proportions. The middle panel highlights individual immune cell populations and the right panel highlights ILCs specifically. (**B** and **C**) Ligand-receptor analysis of spatial transcriptomics data sets for patients with sarcoidosis and non-sarcoidosis patients. Color represents signaling intensity. (**D**) Representative immunohistochemistry (*n* = 7 patients) depicting localization of B cells (CD20, PAX5), T cells (CD3), and macrophages (CD68) in sarcoidosis-affected skin. Dotted box outlines sarcoid granuloma. (**E**) Representative histology (*n* = 3 patients) depicting localization of mature germinal center–like B cells (CD3^–^CD20^+^CD23^+^, white arrows). Scale bars: 50 μm and 5 μm (yellow insets). (**F**) Representative histology (*n* = 3 patients) depicting localization of ILC1 (Lin^–^IL7R^+^Tbet^+^, white arrowheads). Lineage^–^ = CD3^–^CD16^–^CD19^–^CD20^–^CD56^–^CD68^–^ (labeled in green). Scale bars: 100 μm and 10 μm (insets).

We performed spatial deconvolution of individual spots using gene signatures derived from our scRNA-seq data to investigate the distribution of immune cell populations (33). Sarcoid granulomas specifically contained ILCs, B cells, Th1 cells, Th17.1 cells, and mature DCs (Figure 3A and Supplemental Figure 6C). We noted that transcripts of mature differentiated B cell markers, *CR2*, *FCER2*, and *AICDA*, localized specifically within sarcoid granulomas (Supplemental Figure 6D). In contrast, non-sarcoid granulomas exhibited fewer T cells, lacked ILCs completely, and did not express mature differentiated B cell markers (Figure 3A and Supplemental Figure 6D). We performed ligand-receptor analysis using our spatial data sets. Non-sarcoid granulomas exhibited osteopontin (also known as *SPP1*) signaling as the major interaction, and sarcoid granulomas demonstrated prominent *CXCL12/CXCR4* and *CCL19/CCR7* signaling, which are 2 important signaling pathways promoting TLS formation (Figure 3, B and C, and Supplemental Figure 6D). Indeed, *CCL19*, *CCR7*, and *CXCR4* transcripts all localized within sarcoid granulomas (Supplemental Figure 6D).

To complement our spatial transcriptomics data sets, we performed protein immunofluorescence on sarcoidosis-affected skin samples taken from 7 patients (4 patients from our scRNA-seq cohort) (Figure 3D and Supplemental Figure 7). Sarcoid granulomas from all 7 patients displayed distinct aggregates of B cells (Pax5^+^ or CD20^+^) that colocalized with T cells (CD3^+^) and were distinct from macrophages (CD68^+^ or CD163^+^) (Figure 3D and Supplemental Figure 7A). Moreover, many of these B cells were also positive for CD23, an established marker of B cell and TLS maturation (Figure 3E and Supplemental Figure 7B) (34). Finally, ILCs (Lineage^–^CD127^+^Tbet^+^) localized to the periphery of sarcoid granulomas (Figure 3F). Taken together, these results show that B cells and ILCs localized to sarcoid skin granulomas to form mature TLSs.

### Sarcoid granulomas exhibit molecular features resembling mature TLSs.

**Figure 4:**
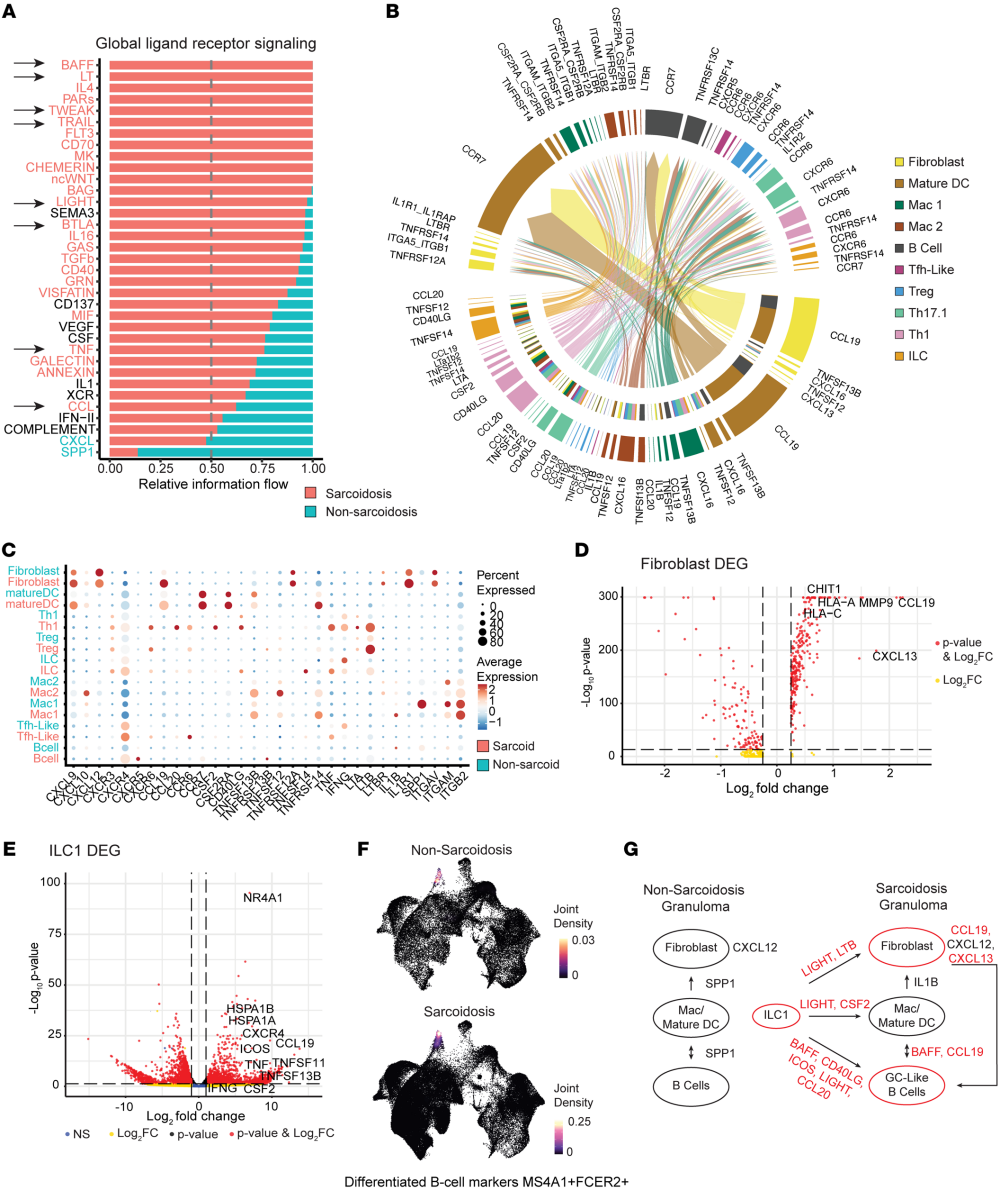
Sarcoid granulomas exhibit molecular features resembling mature tertiary lymphoid structures. (**A**) Global analysis of ligand-receptor pathways between sarcoid and non-sarcoid granulomas. Arrows highlight *TNF* family and *CCL* signaling. (**B**) Cell-specific ligand-receptor analysis. Bottom half of circle depicts secreting cell types, and upper half of circle depicts receiving cell types. Inner bottom circle is a summary of the receiving cell types. (**C**) Dot plot demonstrating average expression (color) and percentage of cells (dot size) expressing specific cytokines. (**D**) Volcano plot of differential gene expression for sarcoidosis and non-sarcoidosis fibroblasts depicting increased *CCL19* and *CXCL13* expression. (**E**) Volcano plot of differential gene expression from bulk RNA-seq of isolated ILC1s from sarcoidosis skin (*n* = 2) vs. sarcoidosis blood (*n* = 2), demonstrating increased expression of *CCL19* and *CXCR4* in skin ILCs. (**F**) Density plot demonstrating that sarcoidosis-specific B cells express *MS4A1* (CD20) and *FCER2* (CD23) markers. (**G**) Summary of unique ligand-receptor interactions found in sarcoid granulomas.

Next, we performed ligand-receptor analysis on our non-sarcoidosis and sarcoidosis single-cell data sets to study the cross-talk among immune cells. Globally, non-sarcoid skin granulomas were dominated by *SPP1* signaling, and sarcoid granulomas were enriched in TNF and CCL family signaling, including B cell–activating factor (*BAFF*), lymphotoxin (*LT*), LIGHT (*TNFSF14*), and *CCL19* (Figure 4A and Supplemental Figure 8A). Cell-specific ligand-receptor analysis revealed that most, if not all, interactions within non-sarcoid granulomas were conserved in sarcoid granulomas, but the reverse was not observed (Supplemental Figure 8, B and C, and Supplemental Table 6). We subtracted the common interaction pairs and focused on 5 sarcoidosis-specific ligand-receptor interactions that promote TLS formation (Figure 4B). (a) A subpopulation of fibroblasts and mature DCs both induced *CCL19*, while mature DCs and B cells both received the signal via *CCR7* (Figure 4, B and C, and Supplemental Figure 8D). (b) Notably, the same subpopulation of fibroblasts also expressed *CXCL13*, which was received via *CXCR5* on B cells specifically (Figure 4, B–D, and Supplemental Figure 8, D and E). (c) ILC1s strongly induced *LIGHT* and *LTB*, individual cytokines previously shown to be sufficient to induce TLS formation (35). LIGHT signals through TNFRSF14 expressed on mature DCs, Mac 1, and Mac 2 cells, and Mac 1 cells induced *IL1B* (Figure 4, B and C). The same subpopulation of fibroblasts received both *IL1B* and *LTB* signals. (d) In addition to LIGHT, sarcoidosis-associated ILC1s also expressed high levels of *CD40L*, *BAFF*, and *ICOS*, which are well-established B cell–activating cytokines (Figure 4E). In fact, prior work demonstrated that ILCs directly activate B cells through BAFF and CD40L (36). Sarcoidosis-associated ILC1s also expressed *CCL20*, which may signal to T helper and B cells through *CCR6*. (e) Finally, sarcoidosis-associated B cells expressed *MS4A1* (also known as CD20), *FCER2* (also known as CD23), *BCL6*, and *AICDA*, which are all markers of late-stage differentiated B cells in mature TLSs (37) (Figure 4F and Supplemental Figure 4B).

Finally, CXCL12/CXCR4, a third signaling pathway important for TLS formation, was strongly induced in both non–sarcoidosis- and sarcoidosis-affected skin (Supplemental Figure 8A) (38). Fibroblasts and macrophages induced *CXCL12*, and multiple immune cell types expressed *CXCR4* (Supplemental Figure 8F). Taken together, these results show that skin granulomas from patients with sarcoidosis and non-sarcoidosis patients exhibited unique molecular differences (Figure 4G). Non-sarcoid granulomas are driven by *SPP1* signaling. In contrast, sarcoid granulomas induce cytokines involved in TLS formation (37).

### Patients with sarcoidosis have increased levels of circulating blood ILC1s.

**Figure 5:**
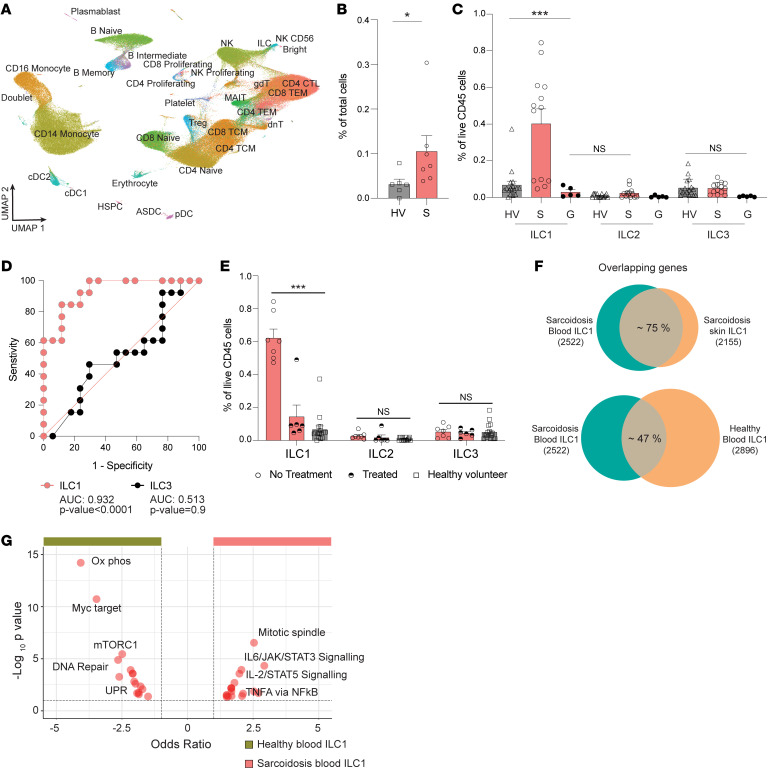
Blood from patients with sarcoidosis contains increased circulating levels of ILC1s. (**A**) Identification of cell clusters from blood of patients with sarcoidosis (S, *n* = 7) and healthy volunteers (HV, *n* = 6). (**B**) Scatter plot shows relative contribution of ILCs as percentage of total cells. Statistical significance was calculated using 2-tailed, unpaired Student’s *t* test. (**C**) Flow cytometry analysis of ILC subtypes as percentage of total CD45^+^ cells in healthy volunteers (*n* = 17), non-sarcoidosis granuloma patients (**G**, *n* = 5), and sarcoidosis patient blood (*n* = 13). One-way ANOVA revealed statistical significance for ILC1: *F*(2,27) = 19, *P < 0.05, ***P < 0.001; ILC2 and ILC3 were not significant among groups. (**D**) Receiver operating characteristic (ROC) curves for ILC1 (red) and ILC3 (black) from sarcoidosis patient PBMCs. Area under the curve (AUC) values are listed. (**E**) Flow cytometry analysis of ILC subtypes as percentage of total CD45^+^ cells in blood from no-treatment patients (*n* = 7), treated sarcoid patients (*n* = 6), and healthy volunteers (*n* = 17). One-way ANOVA revealed statistical significance for ILC1: *F*(2,27) = 55, ***P < 0.001; ILC2 and ILC3 are not significant. (**F**) Comparison of gene expression from bulk RNA-seq analysis of flow cytometry–purified sarcoidosis skin ILC1s (*n* = 2), sarcoidosis blood ILC1s (*n* = 2), and healthy volunteer ILC1s (*n* = 3). (**G**) Volcano plot of pathway analysis comparing flow cytometry–purified ILC1s from sarcoidosis blood and healthy volunteer blood. Data represented as mean ± SEM. NS, not significant. UPR, unfolded protein response; Ox phos, oxidative phosphorylation.

We wanted to assess whether tissue-specific ILC1 changes may also be reflected in circulating blood. We collected peripheral blood mononuclear cells (PBMCs) from 7 patients with sarcoidosis for single-cell sequencing and created a combined approximately 116,767 cell data set (Supplemental Table 1). We also used publicly available data for 6 healthy adult controls (Supplemental Table 2) (Figure 5A and Supplemental Figure 9A). In contrast to skin, we identified cell populations by using a comprehensive single-cell reference atlas created by CITE-seq that is available for human PBMCs (39, 40). Our ILCs overlaid on the ILC coordinates depicted in the atlas (Supplemental Figure 9, B–D). Blood from sarcoidosis patients had an approximately 2.5-fold increase in ILCs compared with healthy volunteers. ILC differential gene expression profile showed a similar induction of inflammatory genes to that seen in ILCs from sarcoid skin (*CCL5*, *GZMA*, and *GZMK*) (Figure 5B, Supplemental Figure 9E, and Supplemental Table 4).

In parallel, we used the same flow cytometry gating strategy to assess ILC subtypes in blood from 13 patients with sarcoidosis, 5 non–sarcoidosis granuloma patients, and 17 healthy controls (Figure 5C and Supplemental Table 1). ILC1 was the major subtype, and healthy volunteers and sarcoidosis patient blood contained, on average, 0.05% and 0.45% of CD45^+^ cells, respectively (Figure 5C and Supplemental Figure 9F). Blood from non–sarcoidosis granuloma patients did not display enrichment of circulating blood ILCs of any subtype. Thus, patients with sarcoidosis exhibited a greater than 8-fold increase in circulating ILC1s. The receiver operating characteristic (ROC) curve assesses the accuracy of a diagnostic test and represents the relationship between the true positive rate (TPR, or sensitivity) of the test and its false positive rate (FPR). The area under the curve (AUC) index for ILC1 and ILC3 was 0.90 and 0.56, respectively (Figure 5D), where a score above 0.7 is considered a reliable biomarker (41). We noted that patients with sarcoidosis exhibited higher and lower populations of circulating ILC1s (Figure 5C). While this difference may reflect the known clinical heterogeneity of sarcoidosis, we subgrouped our sarcoidosis patients based on treatment status (Figure 5E). Patients with active disease that were not receiving any treatment (no-treatment) exhibited 12-fold more circulating ILC1s compared with healthy controls. Patients being actively treated with methotrexate, TNF inhibitors, or hydroxychloroquine had approximately 4-fold less circulating ILC1s compared with no-treatment sarcoidosis patients. The lone patient in the treated group with high ILC1s had active skin lesions despite taking hydroxychloroquine and a PDE4 inhibitor. The difference in circulating ILC1s between treated sarcoidosis patients and healthy controls was not statistically significant. Taken together, these data show that circulating ILC1s at a threshold of 0.45% of CD45^+^ cells may serve as a reliable biomarker for sarcoidosis diagnosis. Circulating ILCs levels may also reflect treatment status.

We also used flow sorting to purify ILC1s from the blood of healthy volunteers and sarcoidosis patients and performed bulk RNA-seq. Using the sarcoidosis blood ILC1 data set as a reference, 75% of the genes overlapped with our sarcoidosis skin ILC1 data set. In contrast, only 47% of the genes overlapped with ILC1s from healthy volunteer blood (Figure 5F). Sarcoidosis blood ILC1s induced genes involved in TNF signaling and the JAK/STAT pathway (Figure 5G). Thus, circulating ILC1s in patients with sarcoidosis showed both increased abundance and signs of increased activation.

### Lung sarcoid tissue also exhibits increased B cells and ILCs.

**Figure 6:**
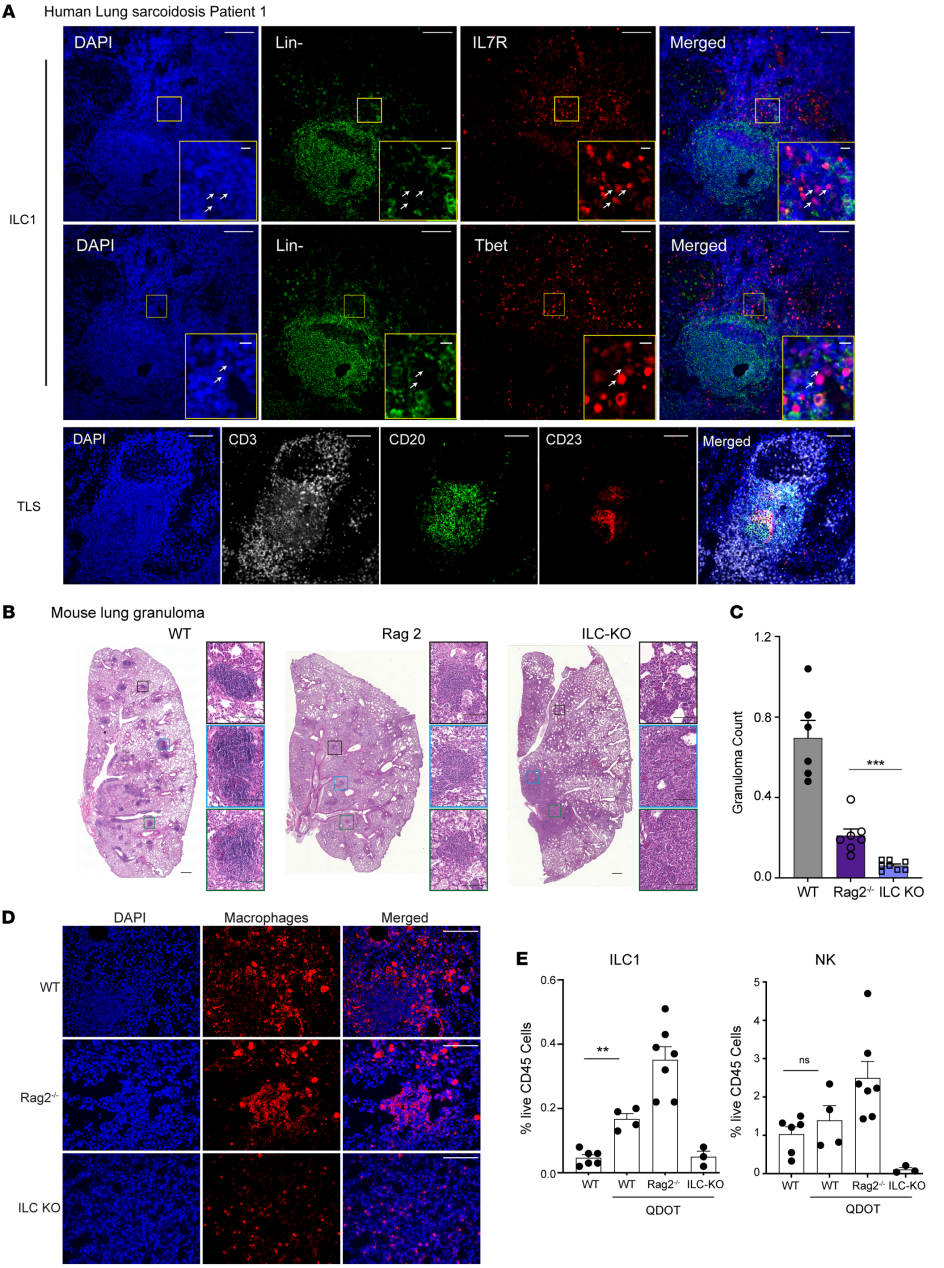
ILCs are necessary for noninfectious granuloma formation in mice. (**A**) Representative histology (*n* = 2 patients) demonstrating ILCs (Lineage^–^IL7R^+^Tbet^+^) and recruitment of B cells (CD20^+^CD23^+^) and to human lung sarcoid granulomas. Lineage^–^ = CD3^–^CD16^–^CD19^–^CD20^–^CD56^–^CD68^–^ (labeled in green). Scale bars: 100 μm and 10 μm (insets). (**B**) Representative H&E staining depicting granuloma formation in whole lung sections from cadmium nanoparticle–treated WT, *Rag2^–/–^*, and *Rag2^–/–^* *Il2rg^–/–^* (ILC-KO) mice. Inset images show higher magnification. Scale bars: 100 μm. (**C**) Quantification of lung granulomas (WT, *n* = 6; *Rag2^–/–^*, *n* = 7; ILC-KO; *n* = 8). (**D**) Representative immunofluorescence depicting macrophage (F4/80^+^) accumulation in lung tissue. Two-tailed, unpaired Student’s *t* test. Scale bars: 100 μm. The WT macrophages are also shown in Supplemental Figure 10C. (**E**) Flow cytometry analysis of ILC1 and NK cells as percentage of live CD45^+^ cells in different mouse genotypes before and after treatment with cadmium nanoparticles (QDOT). WT, *n* = 6; QDOT-treated, WT, *n* = 4; *Rag2^–/–^*, *n* = 7; ILC-KO, *n* = 3. Two-tailed, unpaired Student’s *t* test, comparing WT mice before and after treatment. Data represented as mean ± SEM. **P < 0.01; ***P < 0.001.

We wanted to determine whether sarcoid granulomas in other tissues also recruit B cells and ILCs. Similar to skin granulomas, sarcoid lung tissue displayed distinct aggregates of B cells (CD20^+^) (Figure 6A and Supplemental Figure 10A). Many of these B cells were also positive for CD23, suggesting these B cells were forming mature TLSs (34). Moreover, we found that ILCs (Lineage^–^CD127^+^Tbet^+^) localized to the periphery of sarcoid granulomas (Figure 6A). Taken together, these results show that lung sarcoid granulomas also exhibited increased recruitment of B cells and ILCs.

### ILCs are necessary for noninfectious granuloma formation in mice.

Next, we tested whether ILCs are necessary for noninfectious granuloma formation. There is no broadly accepted mouse model for sarcoidosis. We used the established cadmium nanoparticle (QDOT) mouse model, where pulmonary exposure to QDOT induces formation of lung granulomas (42). Similar environmental exposure to other heavy metals (i.e., beryllium) in humans is also linked to sarcoidosis-like diseases (43). After 30 days, we harvested lung tissue and counted granuloma formation per high-power field across the mid-coronal lung section. We verified that QDOT induced discrete granulomas in WT mice (WT, *n* = 6; QDOT-treated WT, *n* = 4; Figure 6, B–D, and Supplemental Figure 10B). Importantly, these granulomas recruit multiple immune cell types, including T cells, B cells and ILCs, similar to human sarcoid granulomas (Figure 6, D and E, and Supplemental Figure 10, C–F). We performed the QDOT model on mice lacking all ILCs (*Rag2^–/–^* *Il2rg^–/–^*, henceforth known as ILC-KO) as well as control *Rag2^–/–^* mice (8, 44, 45). *Rag2^–/–^* animals may lack mature B and T cells, but these mice remained capable of forming discrete tissue granulomas composed primarily of macrophages (Figure 6, C–E) (46). Notably, ILC-KO mice developed significantly fewer tissue granulomas compared with *Rag2^–/–^* control mice (*n* = 3 and *n* = 7, respectively) (Figure 6, C and D). Finally, we confirmed the absence of ILCs in ILC-KO mice by flow cytometry (Figure 6E and Supplemental Figure 10E). Notably, we did not see increased recruitment of NK cells to QDOT-induced skin granulomas (Figure 6E). We conclude that ILCs are necessary for noninfectious granuloma formation.

### CXCR4/CXCL12 signaling is upregulated in sarcoidosis.

**Figure 7:**
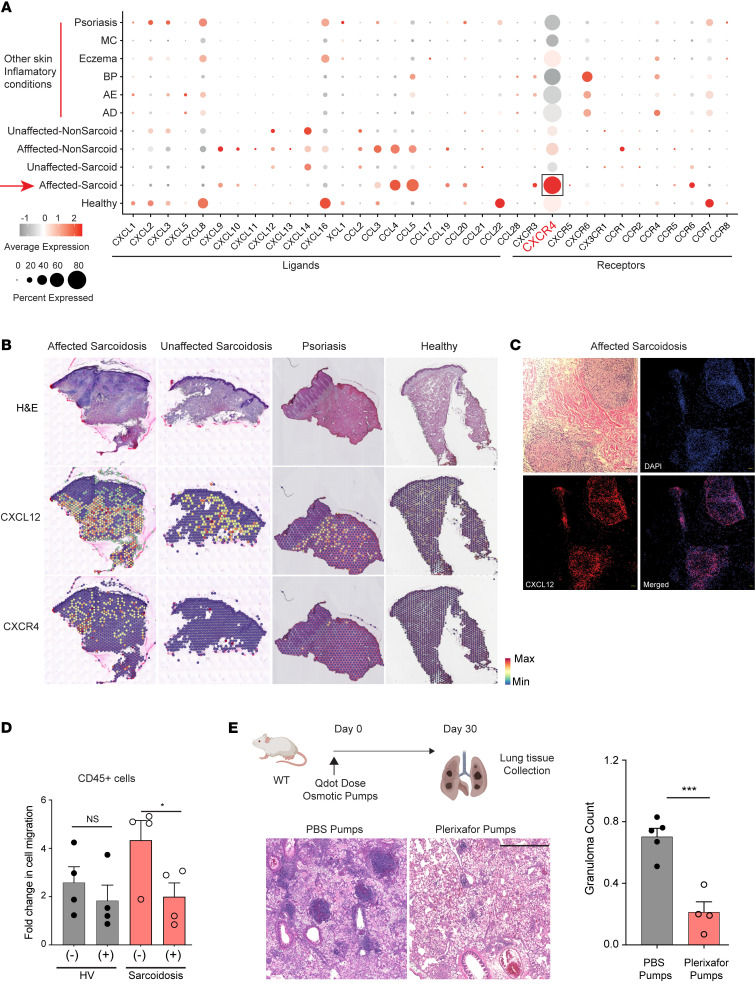
CXCR4 is necessary for ILC1 migration and mouse noninfectious granuloma formation. (**A**) Dot plot comparing cytokine ligand and receptor gene expression in sarcoidosis and skin inflammatory diseases. Dot size reflects percentage cells expressing the gene, and color illustrates level of gene expression. MC, molluscum contagiosum; BP, bullous pemphigoid; AE, acrodermatitis enteropathica; AD, atopic dermatitis. (**B**) Representative spatial transcriptomics depicting *CXCR4* and *CXCL12* expression in affected sarcoidosis (*n* = 2), unaffected sarcoidosis (*n* = 2), psoriasis (*n* = 3), and healthy volunteer skin (*n* = 3). (**C**) Representative immunohistochemistry (*n* = 3) of CXCL12 depicting expression within sarcoid granulomas. Scale bars: 50 μm. (**D**) Fold change in CXCL12-mediated migration of CD45^+^ immune cells from healthy volunteers (HV, *n* = 4) and sarcoidosis blood (*n* = 4) with and without CXCR4 inhibitor (plerixafor). Mean ± SEM. Significance was calculated by 2-tailed, paired Student’s *t* test. (**E**) H&E staining highlighting pulmonary granuloma formation in lung tissue from cadmium nanoparticle–induced mice treated with PBS or plerixafor. Scale bars: 100 μm. Quantification of lung granuloma formation (*n* = 5 in each group). Data represented as scatter plots show mean ± SEM. *P < 0.05; ***P < 0.001 by 2-tailed, unpaired Student’s *t* test.

Multiple immune cell types in sarcoidosis-affected skin exhibited increased expression of TLS-specific pathways, including CXCL12/CXCR4 signaling. Compared with gene expression profiles of other inflammatory skin diseases, sarcoidosis induced the strongest global expression of *CXCR4* (Figure 7A) (32, 47, 48). Consistently, spatial transcriptomics localized *CXCR4* expression within sarcoid granulomas, and expression was minimal or not seen in patients with psoriasis or healthy unaffected skin (Figure 7B and Supplemental Figure 11) (49). Moreover, protein immunofluorescence confirmed strong CXCL12 expression within the center of sarcoid granulomas (Figure 7C).

In addition to promoting TLS formation, CXCL12/CXCR4 signaling has been shown to regulate immune cell migration (50). To test whether CXCR4 regulates sarcoidosis-activated immune cell function, we performed a migration assay with PBMCs isolated from healthy volunteers and patients with sarcoidosis. In response to its cognate ligand CXCL12, CD45^+^ circulating immune cells from sarcoidosis patients exhibited approximately 2-fold greater cell migration compared with CD45^+^ immune cells from healthy volunteers (Figure 7D). When CD45^+^ circulating immune cells were treated with a well-established pharmacologic CXCR4 inhibitor, plerixafor (also known as AMD3100), they no longer exhibited improved migration compared to immune cells from healthy volunteers (Figure 7D). We conclude that the increased expression of CXCR4 on sarcoidosis-activated immune cells improved cell migration toward CXCL12.

We returned to the QDOT granuloma model to test whether CXCR4 signaling is necessary for noninfectious granuloma formation. Mice were administered plerixafor or vehicle control by osmotic pumps. Compared with control-treated mice, plerixafor-treated WT mice developed fewer QDOT-induced lung granulomas (*n* = 5 in control group; *n* = 4 in plerixafor-treated group) (Figure 7E). Taken together, these results show that CXCR4 signaling is necessary for formation of noninfectious tissue granulomas.

## Discussion

Our results directly address 2 longstanding questions in the sarcoidosis field, the discovery of a disease biomarker and the identification of an actionable treatment target. We also demonstrated that formation of skin granulomas is not a generic molecular response, and granulomas from different diseases exhibit unique cellular and molecular characteristics. Non-sarcoid granulomas were dominated by macrophages and *SPP1* signaling. In contrast, sarcoid skin granulomas recruited B cells and ILC1s specifically and exhibited unique *CXCR4*, *CXCL13*, and *CCL19* signaling to form mature TLSs.

Sarcoidosis is a clinically heterogeneous disease. Because there are no biomarkers specific for sarcoidosis, current diagnosis requires active exclusion of other potential diseases, which takes time and delays treatment. Our data demonstrate that tissue and circulating ILC1s are elevated in sarcoidosis compared with non-sarcoidosis skin granulomatous diseases and may serve as a reliable biomarker. Indeed, tuberculosis is a granulomatous disease that ranks at the top of alternative diagnoses for sarcoidosis, and prior studies demonstrated that active tuberculosis patients exhibit decreased circulating ILCs compared with healthy controls (12). Our sarcoidosis single-cell data set was larger than previously published studies and included non-sarcoidosis skin granulomas as an additional control, which may explain our identification of ILCs. Future prospective studies are needed to test new patients suspected with sarcoidosis in any organ and measure tissue and circulating ILC levels. If successful, a tissue or blood test would speed up diagnosis as well as potentially help patients avoid high-risk tissue sampling if suspected sarcoidosis is limited to organs such as the heart or brain. Measuring ILC1s currently requires high-resolution flow cytometry that is not widely available. However, ILC1s would be the first effective sarcoidosis biomarker, and these results will undoubtedly motivate future efforts to develop better tools to detect ILC1s on lower-resolution systems.

Circulating levels of ILC1s in sarcoidosis also correlated with treatment status. Monitoring sarcoidosis disease activity currently requires serial imaging scans, exposing patients to risks of cumulative radiation exposure. These scans are necessary to determine when to stop immunosuppression with oral corticosteroids. A blood test to assess disease activity would minimize radiation exposure as well as shorten exposure to the significant side-effects of oral corticosteroids. A future clinical trial could prospectively follow sarcoidosis patients, measure ILC1s in blood, and quantitatively grade multiorgan disease activity to test the clinical applicability of our results.

Similar to our human lung sarcoidosis data, we showed that cadmium nanoparticles generate pulmonary granulomas that replicate sarcoid granulomas in terms of immune cell recruitment, including B cells and ILCs. We used this model to show that ILCs are necessary for noninfectious granuloma formation. Our single-cell data suggest direct and indirect pathways where ILC1s may contribute to TLS and granuloma formation. Sarcoidosis-activated ILC1s express *CD40L*, *ICOS*, and *BAFF*, which were previously shown to mediate direct interactions between ILCs and B cells (51, 52). Sarcoidosis-activated ILC1s also express *LIGHT*, which was sufficient to induce local TLS formation (35). Thus, ILCs may directly promote TLS formation and sarcoidosis-like granuloma formation. However, ILCs may also directly affect macrophage behavior and subsequent granuloma formation, because *Rag2^–/–^* mice lacking B cells are still capable of generating discrete granulomas, and ILC-KO mice lacking B cells and ILCs form significantly fewer granulomas. We recognize the technical limitations within the ILC1 field, including the lack of a specific ILC-knockout mouse (without B and T cells being affected), a more specific ILC1-knockout mouse, a purified ILC1 cell line, and ILC1-specific clinical therapeutics. Additional development of new molecular tools will permit dissecting the specific role of ILC1s in noninfectious granuloma formation. As an alternative strategy, we used existing tools to target sarcoidosis-specific molecular signals.

Our single-cell analysis revealed that sarcoid skin granulomas specifically induced TLS-forming signaling pathways, including *CXCL12/CXCR4* signaling. Recent analysis of neurosarcoidosis cerebrospinal fluid and blood samples also identified increased CXCR4 signaling (53). Activated CXCR4 is a G protein–coupled receptor that signals through the JAK/STAT pathway, specifically JAK2/JAK3 (54). Inhibitors of JAK/STAT signaling are currently being tested in patients with refractory sarcoidosis (30, 55, 56). However, their use is associated with well-documented serious side-effects, including cardiac complications, blood clots, infection, and cancer. We showed that a CXCR4 inhibitor, plerixafor, attenuated granuloma formation in a mouse model.

Plerixafor (AMD3100) is an FDA-approved small molecule inhibitor of CXCR4 that is used acutely in the clinic to mobilize stem cells for bone marrow transplantation, and chronic use is restricted because at 16-times higher than the FDA-approved dose, cardiac-related symptoms appear (57). Plerixafor was recently successfully used at lower doses in 6-month intervals in patients with warts, hypogammaglobulinemia, infections, and myelokathexis (WHIM) syndrome, a genetic disease defined by gain-of-function mutations in CXCR4 (58, 59). Repurposing existing drugs offers a potentially more rapid pathway to deployment since the safety profiles are known. Our data support new clinical trials that test 3–6 months courses of plerixafor in patients with sarcoidosis as a new targeted and rational therapy.

Finally, recent work demonstrating the prognostic value of TLSs in chronic inflammatory diseases has strengthened interest in these structures as potential therapeutic targets. While B cells have previously been reported within sarcoid granulomas, their role has not been well studied. Notably, serum levels of BAFF were found to correlate with sarcoidosis disease activity, and the anti-CD20 monoclonal antibody rituximab has shown some clinical efficacy in patients with refractory sarcoidosis (60, 61). More work is needed to determine whether targeting or reversing TLS formation through B cells directly or TLS-specific signals will prevent granuloma formation or disrupt existing granulomas.

In summary, integration of cellular and molecular data across skin and blood helped identify a tissue and circulating immune cell biomarker as well as a new potential target for treatment. The ability to directly interrogate blood and skin allows this approach to be generalizable to other systemic inflammatory disorders.

## Methods

### Sex as a biological variable

To address sex as a biological variable, we attempted to recruit equal numbers of male and female patients, and these numbers are indicated in the main text when appropriate. We performed all animal experiments with equal numbers of male and female mice.

### Experimental model and participant details

#### Study participants (human).

Skin granulomatous disease diagnosis was confirmed by a team of dermatopathologists and clinicians based on histological assessment, patient history, and clinical phenotype. The patient data and associated demographics are provided in Supplemental Table 1. Demographic information was provided by the participants, and the options were defined by the investigators. Punch biopsies (4–6 mm) were taken from affected skin and unaffected skin. Healthy control skin samples were deidentified from discarded tissue obtained from the Skin Biology and Disease Resource Center (SBDRC) at the University of Pennsylvania and used for flow cytometry. All biopsies were placed on saline-soaked gauze prior to processing. For patient PBMCs and plasma, whole blood samples were collected in collection tubes with EDTA (Becton Dickinson) to prevent clotting. Healthy volunteer PBMCs and plasma were obtained from the Human Immunology Core (HIC) at the University of Pennsylvania. Four deidentified sarcoidosis skin tissue sections were obtained from the Skin Biology and Disease Resource Center (SBDRC) at the University of Pennsylvania, and this tissue was used specifically for immunohistochemistry and immunofluorescence (labeled as sarcoidosis patients A–D) (Supplemental Table 1).

#### Mouse models.

C57BL/6J (stock 000664), *Rag2/Il2rg* (C;129S4-*Rag2^tm1.1Flv^ Il2rg^tm1.1Flv^/*J, stock 014593), and *Rag2* (C57BL/6J-*Rag2^em3Lutzy^*/J, stock 033526) mice were purchased from The Jackson Laboratory. All mice were group housed in the animal facility of the University of Pennsylvania on a 12-hour light/12-hour dark cycle with ad libitum access to water and normal chow. For the in vivo granuloma model, mouse lungs were instilled with a single dose of Qdot (40 μL) (Q21361MP, Thermo Fisher Scientific) particles (42). Thirty days later, mice were sacrificed and lung tissues were harvested for analysis. For mice on plerixafor, Qdot and osmotic pumps containing plerixafor were implanted simultaneously in the mice. Pumps were implanted subcutaneously into the back of the mouse. The pump released approximately 8 mg of AMD3100 per kilogram of body weight per day (62). The control pumps were filled with PBS.

#### Human tissue processing, scRNA-seq library preparation and sequencing.

The skin punch biopsies were incubated in 200 μL of Dispase solution (2 mg/mL; D4818, Sigma-Aldrich) for 30 minutes at 37°C. Following the incubation, the epidermis was peeled from dermis with curved forceps, washed in PBS, and successively minced into <1 mm^3^ pieces, using a scalpel in a serum-free RPMI 1640 media with DNase I (0.2 mg/mL, 12633012, Thermo Fisher Scientific), 20 mM HEPES, and 0.25 mg/ml Liberase (5401119001, Roche). The suspension was incubated for 90 minutes at 37°C. The digestion was stopped by adding 100 μL FBS and 3 μL of 0.5 M EDTA and filtered through a 70-μm cell strainer (22-363-548, Thermo Fisher Scientific). The cells were pelleted and washed twice with PBS containing 1% BSA. Finally, cells were resuspended in PBS containing 0.04% BSA and an aliquot was taken for counting. The scRNA-seq was performed using 10× Chromium 3 v3.1 kit (1000268, 10× Genomics). The sequencing libraries were prepared per manufacturer’s protocol and sequenced using 2 × 100-bp paired-end runs on Illumina HiSeq 2000/HiSeq 2500 platforms at BGI America. The raw and processed sequencing data details are given in Supplemental Table 2.

#### Bulk RNA library preparation.

Flow-sorted cells were lysed immediately by adding TRIzol LS reagent (Thermo Fisher Scientific, 10296010). The samples were vortexed for 20 seconds, 0.2× volumes of chloroform was added, tubes were mixed by inverting, and samples were centrifuged at 16,000*g* at 4°C for 15 minutes. The aqueous phase was then purified and the RNA-seq libraries were made using the NEBNext Single Cell/Low Input RNA Library Prep Kit (E6420S/L, New England Biolabs), following the manufacturer’s instructions.

#### Histology and immunohistochemistry.

Standard histology and immunostaining protocols were performed, and investigators were blinded to tissue origin during histologic staining. In brief, the fresh skin tissue was fixed overnight at 4°C in 4% paraformaldehyde (J19943-K2, Thermo Fisher Scientific). Full-thickness skin was removed from the mouse onto a paper towel. The skin was fixed by inverting the paper towel onto the surface of the fixative (4% paraformaldehyde in PBS), and incubated overnight at 4°C. The following day, the skin was trimmed, placed into tissue cassettes, processed (VIP5b, Sakura) and embedded into wax (Leica Paraplast X-tra) blocks. Blocks were cut using disposable blades (D554P, Sturkey) on a rotary microtome (RM2235, Leica) set at 5 μm thickness. Sections were floated on a water bath (145702, Boekel) set at 43°C and collected onto positively charged glass slides (Fisherbrand Superfrost Plus). Following overnight drying at room temperature, slides were baked for 30 minutes at 60°C, followed by H&E staining using an automated stainer (Leica Autostainer XL). Slides were processed by the Skin Biology and Disease Resource–Based Core (SBDRC) at the Department of Dermatology, University of Pennsylvania, who performed H&E staining of the slides. H&E-stained sections were examined by a board-certified dermatopathologist under bright-field microscopy. For human TLS immunofluorescence microscopy, antibodies against the following proteins were used: CD3 (MCA1477, Bio-Rad), CD20 (14-0202-82, Thermo Fisher Scientific), and CD23 (NB120-16702, Novus Biologicals). For Figure 7C, anti-CXCL12 (MAB350, R&D Systems) was used. For human TLS immunohistology, antibodies against the following proteins were used: CD3 (PA0553, Leica), CD20 (IR604, DAKO), CD68 (PA0273, Leica), CD163 (163M-18, Cell Marque), and PAX-5 (610863, BD Biosciences). For human ILC1 identification, antibodies against the following proteins were used. Lineage^–^ channel: CD3 (MA5-12577, Thermo Fisher Scientific), CD19 (LE-CD19, Bio-Rad), CD68 (MA5-13324, Thermo Fisher Scientific), CD56 (LS-B5569, LSBio), CD16 (88251, Cell Signaling Technology), and CD20 (14-0202-82, Thermo Fisher Scientific). ILC1^+^ marks: Tbet (97135, Cell Signaling Technology) and CD127 (LS-B14308, LSBIO). For ILC1 staining, 2 sequential sections were stained with anti-Lineage antibodies and either anti-Tbet or anti-CD127. To highlight the presence of ILC1, we focused on regions that have cells negative for Lineage marks and positive for either CD127 or Tbet in the same vicinity. For mouse granuloma detection, antibodies against the following proteins were used: for macrophages, a pool of F4/80 (70076, Cell Signaling Technology), CD163 (68922, Cell Signaling Technology), and CD80 (8679, ProSci); for T cells, CD3 (14-0032-82, Thermo Fisher); for B cells, B220 (MCA1258G, Bio-Rad).

Similarly to skin, lung tissues were collected from mice and were fixed overnight at 4°C in 4% paraformaldehyde solution. Lung samples were processed for sectioning by the SBDRC. The SBDRC prepared formalin-fixed, paraffin-embedded slides and performed histology on mid-sagittal sections of lungs. Histology slides were imaged using tiled imaging at ×10 magnification on a Leica DM6B/DMC2900 imaging system (Leica Microsystems). Granulomas were counted for each ×10 field and averaged over a total number of ×10 fields per lung. The granuloma score for lung tissue was calculated as number of granulomas per ×10 field.

#### PBMC and plasma isolation.

Blood from Vacutainers was collected in 50 mL conical tubes, and an equal volume of HBSS (21-023-CV, Corning) was added to each sample. The blood was then poured gently over Ficol-paque (17-140-02, GE Healthcare) at a 1:1 ratio by volume. The tubes were centrifuged at 400*g* and 10°C for 25 minutes with acceleration and deceleration set to 0. The plasma and PBMCs were carefully aspirated. The PBMCs were washed with PBS and aliquots were stored in freezing media (10% DMSO in FBS) in 2 mL cryovials. The cryovials were transferred to –80°C for 24 hours before storing in liquid nitrogen.

#### Spatial transcriptomics.

Spatial transcriptomics was performed on 3 patient samples using the Visium Spatial Gene Expression Slide & Reagent kit (1000187, 10× Genomics). The tissue permeabilization conditions were optimized using the Visium Spatial Tissue Optimization Slide & Reagent kit (1000193, 10× Genomics). Fresh skin punch biopsies were frozen in OCT blocks and stored at −80°C until sectioning. For each patient, 2 sections of lesional tissue and 1 section of nonlesional tissue were taken at 10 mm thickness. The sections were then mounted onto capture areas marked on Visium slides. All the steps including H&E staining of the slides, bright-field image capture, tissue permeabilization (30 minutes), cDNA amplification (16 cycles), and library generation were done following the manufacturer’s protocol. The libraries were sequenced in house using the recommended 28-10-10-120 cycle read setup on the Illumina HiSeq 2500 platform. The sequencing data were mapped to the GRCh38 reference genome using the spaceranger pipeline (v1.3.0, 10× Genomics) to generate gene count and cell barcode matrices. The raw and processed sequencing data details are given in Supplemental Table 1. The histology images were acquired using a Leica DM6B/DMV2900 imaging system (Leica Microsystems). The psoriasis and healthy volunteer spatial data sets were downloaded (49). The psoriasis section represented in Figure 7B corresponds to the section ST 16_Lesional in the original data set and represents a moderate to severe psoriatic phenotype. The sections presented in Supplemental Figure 11B correspond to the sections ST_13_Lesional and ST_17_Lesional in the original data set and represent a mild psoriatic phenotype. The spatial plots for Figure 7B and Supplemental Figure 11B were generated using Seurat workflow.

#### Flow cytometry analysis of ILCs.

Single-cell suspensions were prepared from PBMCs and skin biopsies. ILCs were characterized by flow cytometry gating strategy as previously described (12). Cells were stained with a near-infrared cell viability dye (423105, Zombie NIR, BioLegend) for 15 minutes in the dark at room temperature. Cells were pretreated with Human TruStain FcX Fc-blocking agent (422302, BioLegend) and subsequently stained with monoclonal antibodies against the following proteins: CRTH2 (350104, clone BM16, BioLegend), CD127 (351320, clone HIL-7R-M21, BD Biosciences; clone A019D5, BioLegend), CD117 (313215, clone 104D2, BioLegend), CD56 (318335, clone HCD56, BioLegend), CD94 (562361, clone HP-3D9, BD Biosciences), CD161 (339915, clone HP-3G10, BioLegend), CD16 (302048, clone 3G8, BioLegend), CD4 (300552, clone RPA-T4, BioLegend), and CD45 (304035, clone HI30, BioLegend). Lineage markers: CD19 (clone HIB19, BioLegend), CD34 (clone 561, BioLegend), CD11c (337213, clone Bu15, BioLegend), CD14 (clone HCD14, BioLegend), CD4 (317408, clone OKT4, BioLegend), TCRαβ (306705, clone IP26, BioLegend), TCRγδ (331207, clone B1, BioLegend), BDCA2 (354207, clone 201A, BioLegend) and FcER1 (clone AER-37 [CRA1], BioLegend). Intracellular staining was done after using a Fix/Perm kit (BD Biosciences) and included anti-CD3 (300450, clone UCHT1, BioLegend). Samples were acquired on a 4-laser BD LSRII flow cytometer and all sample data were analyzed using FloJo software version 10.8.1 (BD Biosciences).

For mouse ILC detection, single-cell suspensions were prepared from mouse lung tissues. ILCs were characterized by flow cytometry using a gating strategy as previously described (63). Cells were stained with a cell viability dye (65-0865-14, Thermo Fisher Scientific) for 15 minutes in the dark at room temperature. Cells were pretreated with Mouse TruStain FcX Fc-blocking agent (101320, BioLegend) and subsequently stained with monoclonal antibodies against the following proteins: CD49b (108912, clone DX5, BioLegend), NK1.1 (56-5941-82, clone PK136, Thermo Fisher Scientific), NKp46 (56-3351-82, clone 29A1.4, Thermo Fisher Scientific), CD45 (103137, clone 30-F11, BioLegend), CD49a (564863, clone Ha31/8, BD Biosciences), Lineage markers CD3E (100306, clone 145-2C11, BioLegend), TCRb (109205, clone H57-597, BioLegend), TCRγδ (118105, clone GL3, BioLegend), Ter119 (116205, clone TER-119, BioLegend), Gr-1 (127605, clone 1A8, BioLegend), CD19 (115505, clone 6D5, BioLegend), and B220 (103205, clone RA3-6B2, BioLegend). Samples were acquired on a 5-laser BD LSRFortessa flow cytometer and all sample data were analyzed using FloJo software version 10.8.1 (BD Biosciences).

#### Chemotaxis Transwell assay.

Chemotaxis assays were performed using 6.5 mm, 5 μm Transwell inserts (3421, Corning). PBMCs (500,000) were washed and plated on the upper chamber of the Transwell in 200 μL RPMI 1640 medium containing 0.5% serum (low-serum media). The bottom chamber of the negative control wells contained 500 μL of low-serum media. The other chambers contained 500 μL of low-serum media with CXCL12 (final concentration 100 ng/mL; 300-28A, PeproTech). After incubation for 2 hours at 37°C in a CO_2_ incubator, cells in the bottom chamber were collected and processed for ILC1 quantification by flow cytometry. For each condition, 4 wells (2 × 10^6^ PBMCs) were plated, and after migration, the cells were pooled for flow cytometry analysis. For plerixafor (AMD3100; A5602, Sigma-Aldrich) inhibition of CXCR4 receptors, the PBMCs were first incubated with plerixafor (final concentration 1 μM) for 1 hour at 37°C in a CO_2_ incubator. Following plerixafor incubation, the cells were washed and then proceeded to chemotaxis assay. We reported our results as a fold change of the cell migration. The fold change was calculated as a ratio of the number of cells migrated in response to CXCL12 to the number of cells migrated without CXCL12 within the same sample. This would control for the possibility of different starting numbers in different samples.

### Computational and statistical analysis

#### scRNA-seq data analysis.

The scRNA-seq data were mapped to the GRCh38 reference genome to generate gene count and cell barcode matrices using the cellranger count function from the CellRanger pipeline (v5.0.1, 10× Genomics). All downstream analysis steps were performed using the R package Seurat (64) (v4.3.0, https:// github.com/satijalab/seurat) unless otherwise noted. In brief, Seurat functions Read10X and CreateSeuratObject were used to import and create a merged Seurat object from all filtered feature barcode matrices generated by the CellRanger pipeline. Cells with less than 250 genes, less than 500 unique molecular identifiers (UMIs), less than 0.80 log_10_ genes per UMI, and more than 20% mitochondrial reads were excluded from the merged Seurat object for further analysis. Genes that were detected in less than 10 cells were also discarded. DoubletFinder was used to identify potential cell doublets as a final quality control (65). To determine and regress out the effect of cell cycle, each cell was given a cell cycle phase score using the Seurat function CellCycleScoring (66). The individual data sets were then log-normalized and scaled by linear regression against the number of reads. The FindVariableFeatures function followed by SelectIntegrationFeatures function (nfeatures = 3000) were used to identify variable genes from each individual Seurat object. For cross-tissue data integration and batch correction, FindIntegrationAnchors and IntegrateData were applied to the individual sample Seurat object. Following sample integration, dimensionality reduction was performed using the RunPCA and RunUMAP function–generated UMAP plots. Next, Louvain clustering was performed with the FindClusters function using the first 40 principal components (PCs) and at a resolution of 1.4. We used the ElbowPlot function in Seurat, visual inspection of DimHeatmap plots at different dimensions, and R package clustree to choose an optimal number of dimensions and resolution.

#### Cell type annotation.

We used 3 complementary approaches to annotate the identities of different cell clusters: (a) we checked the expression of lineage-specific marker genes identified from previously published scRNA-seq studies in our query cluster marker genes list and in differentially expressed genes of the query cluster. (b) We applied an unbiased cell type recognition method named deCS (R package) (67), which leverages mapping of the top 100 genes from the query cluster to the reference transcriptomic data sets of known cell types such as BlueprintEncode (68), MonoccoImmune reference (69), and Database of Immune Cell Expression (DICE) data (70). We first applied deCS to determine whether the predicted annotations were consistent with our findings and then assigned the identity to the cluster. (c) For the PBMC scRNA-seq data set, we annotated our clusters by using the Seurat reference mapping function to overlay our gene expression profiles onto the multimodal PBMC atlas. The reference mapping function was also employed to assist in the identification of the immune cell population from the skin scRNA-seq data set from sarcoidosis and granuloma immune cell subclusters. The sample statistics and marker gene dot plots were made by using dittoSeq (v1.4.1; https://bioconductor.org/packages/release/bioc/html/dittoSeq.html). UMAP was applied to visualize the single-cell transcriptional profile in 2D space based on the SNN graph described above (71). Other bar plots, box plots, violin plots, and heatmaps were generated by customized R code through ggplot2 (v3.2.1, R package) (72).

#### CellChat.

We used the R package CellChat (v1.5.0) to study the ligand-receptor interaction networks between different immune cell subclusters (73). We performed the ligand-receptor interaction analysis on the immune subcluster from the sarcoidosis scRNA-seq data set. The analysis was performed twice, once with all ligand interaction pairs and second on the paracrine signaling network. For our analysis, we considered ligand-receptor interactions that were expressed in at least 10 cells. The CellChat algorithm calculates an aggregated ligand-receptor interaction score base on a method called “trimean.” The CellChat algorithm has the added advantage of comparing 2 or more single-cell data sets and gives a comparative score for the given cell types. These scores represent the probability of interaction among the ligand-receptor pairs. The probability was then visualized using functions such as netAnalysis_signalingRole_scatter, which visualizes the major sender and receiver across all cell types, and netAnalysis_signalingChanges_scatter, which identifies the major signaling networks acting within a given cell type.

#### Bulk RNA-seq analysis.

Fastq files were aligned to the hg19 reference genome using STAR_2.4.0 in basic 2-pass mode using the Encode options as specified in the manual. Reads overlapping with annotated genes (Ensembl build hg19) were counted using the summarizeOverlaps function from the R package GenomicAlignments in strand-specific, paired-end mode. Fragments per kilobase per million mapped fragments (FPKM) counts and differential expression was estimated using DESeq2 (https://bioconductor.org/packages/release/bioc/html/DESeq2.html).

#### Spatial transcriptomics.

Following the mapping of spatial RNA-seq data, Seurat and Giotto software were used to analyze the data. In brief, Seurat was used to load the spatial data and the SpatialFeaturePlot function was used to plot the number of nUMI (nCount_Spatial) and number of genes (nfeature_Spatial). The individual samples were then normalized using SCTransform. To generate gene plots of key genes, the interactive plotting feature SpatialDimplot was used from the Seurat pipeline. To identify the differences in spatial distribution of immune cells in unaffected and affected sections, the cell-gene matrix and spatial coordinates were analyzed using Giotto (33). Briefly, the cell-gene matrix and cell spatial coordinates were processed to create a Giotto object. After image alignment, the Giotto object was filtered for genes detected in a minimum number of cells (cutoff = 5) and minimum genes detected per cell (cutoff = 100). The filtered object then underwent normalization, dimensional reduction, and clustering. We used default parameters to find the spatial distribution of genes using a ranking method. The top 100 genes were used for fGSEA analysis. The associated Gene Ontology (GO) enrichment and Kyoto Encyclopedia of Genes and Genomes (KEGG) pathways for each cluster for every sample are given in Supplemental Table 3. For cell type annotation, we used dampened weighted least squares–based deconvolution where the signature matrix used for deconvolution was derived from the same patient’s scRNA-seq data set.

#### Functional enrichment analysis.

The GO representation analysis and KEGG pathway enrichment analysis were performed using R packages clusterProfler (v3.18.1) and enrichplot (v1.10.2; https://bioconductor.org/packages/release/bioc/html/enrichplot.html). GO/KEGG pathway terms with adjusted *P* values of less than 0.05 were regarded as statistically significant.

### Statistics

Presented data combine all experiments, and unless noted, all experiments were repeated 2–3 times independently. Experiments were not randomized, and investigators were not blinded to allocation during experiments and outcome assessment, unless noted in the text. Comparisons between 2 groups were carried out using Student’s *t* test and between multiple groups were carried out using ANOVA. For correlated data, paired *t* tests were used for 2 groups and within-subject ANOVA were used for multiple groups. In all tests, a *P* value of less than 0.05 was considered significant, with higher levels of significance indicated as **P < 0.01 and ***P < 0.001 in the text. When appropriate, specific *P* values are provided in figure legends.

### Study approval

Human patients diagnosed with skin granulomatous diseases were recruited to the study at Perelman Center for Advanced Medicine, University of Pennsylvania. Written informed consent was obtained before participation in the study under a protocol approved by the IRB of the University of Pennsylvania School of Medicine (IRB 832147).

Experiments involving mice were reviewed and approved by the Institutional Animal Care and Use Committee of University of Pennsylvania (protocol 805620). Mice were treated in accordance with the NIH *Guide for the Care and Use of Laboratory Animals* (National Academies Press, 2011).

### Data availability

Underlying data can be accessed through the Supporting Data Values file. All sequencing data have been deposited in the NCBI Gene Expression Omnibus (GEO) and are publicly available as of publication. (a) GSE227041 (https://www.ncbi.nlm.nih.gov/geo/query/acc.cgi?acc=GSE227041 Token: cpwbaucadbavdun). (b)GSE226896 (https://www.ncbi.nlm.nih.gov/geo/query/acc.cgi?acc=GSE226896 Token: wfcpyekutpufjsr).

This paper does not report any original code. Any additional information required to reanalyze the data reported in this paper is available from the lead contact upon request.

## Author contributions

Clinical sample collection was performed by MR. Experiment conceptualization, methodology, and investigation was performed by SS, PJ, CM, AEK, JTS, JH, MLH, SMP, and THL. Bioinformatics analysis was performed by SS, VJ, and SGG. Statistical analysis was performed by DHL. THL and SS wrote the manuscript, which was reviewed and edited by MR, SS, and OA.

## Supplementary Material

Supplemental data

Supplemental table 1

Supplemental table 2

Supplemental table 3

Supplemental table 4

Supplemental table 5

Supplemental table 6

Supporting data values

## Version 1 09/03/2024

Electronic publication
