# Supplementary material for: Recruitment of CXCR4+ type 1 innate lymphoid cells distinguishes sarcoidosis from other skin granulomatous diseases
Source: J Clin Invest. 2024 Sep 3;134(17):e178711. doi: 10.1172/JCI178711 (PMC11364400; doi:10.1172/JCI178711)
Supplement: Supplemental data [file jci-134-178711-s157.pdf]

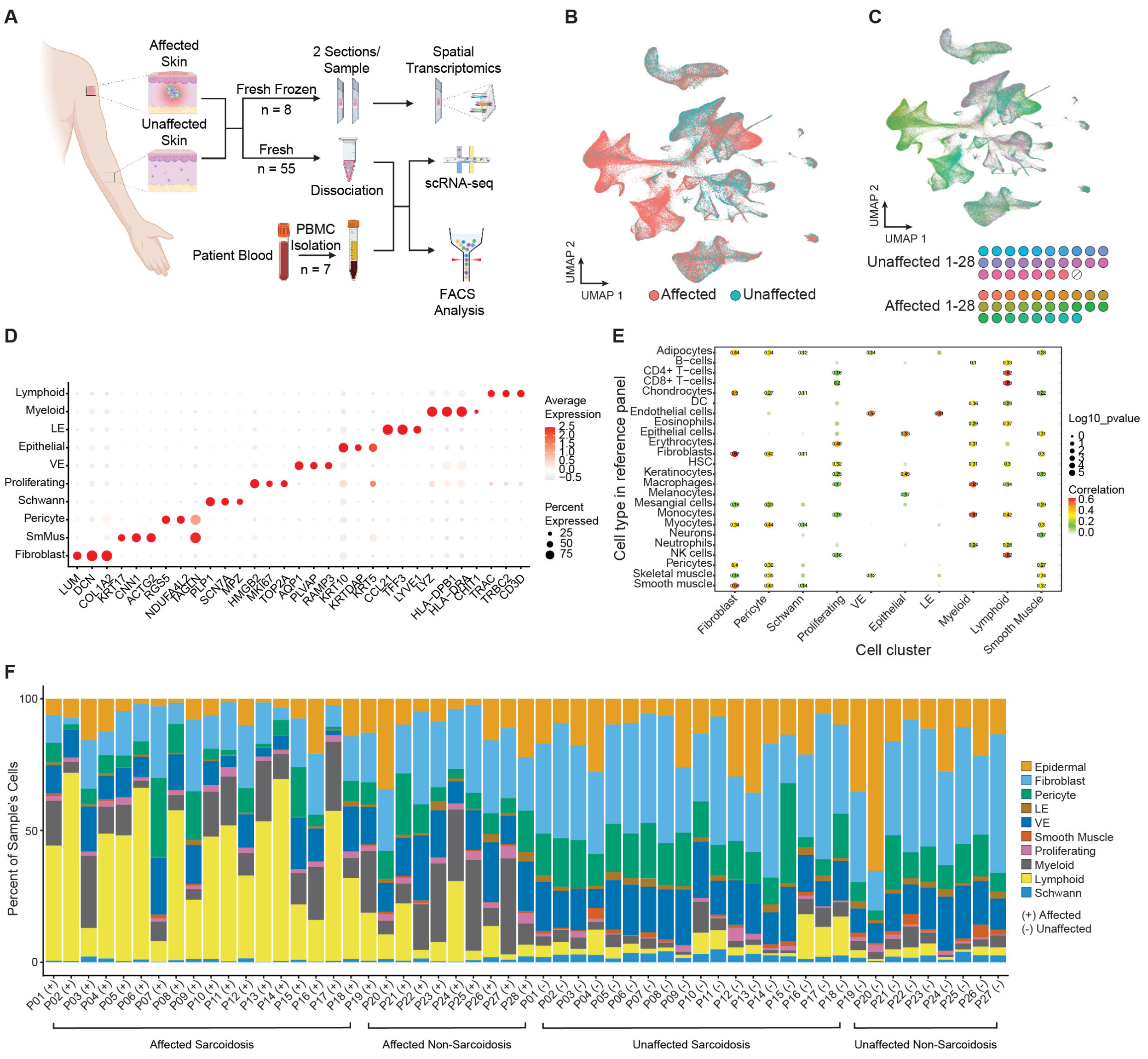

Figure S1: Single-cell analysis of human skin granulomas.
(**A**) Summary of analysis strategy. (**B-C**) UMAP plots of unaffected and affected skin granuloma samples (N=55). Individual patient samples are depicted in C. (**D**) Dot plot of key marker genes for each cell type. Color scale represents gene expression, and dot size represents percentage of cells expressing the marker gene. (**E**) deCS correlation plot for cell type enrichment analysis of different cell clusters. Y-axis: main cell types identified from BlueprintEncode database. Color scale represents Pearson correlation coefficient, and dot sizes represent the -log10 transformed p value. (**F**) Bar plot showing relative contribution of different cell types in individual skin samples. VE, Vascular endothelium; LE, lymphatic endothelium; VSMC, vascular smooth muscle cells.

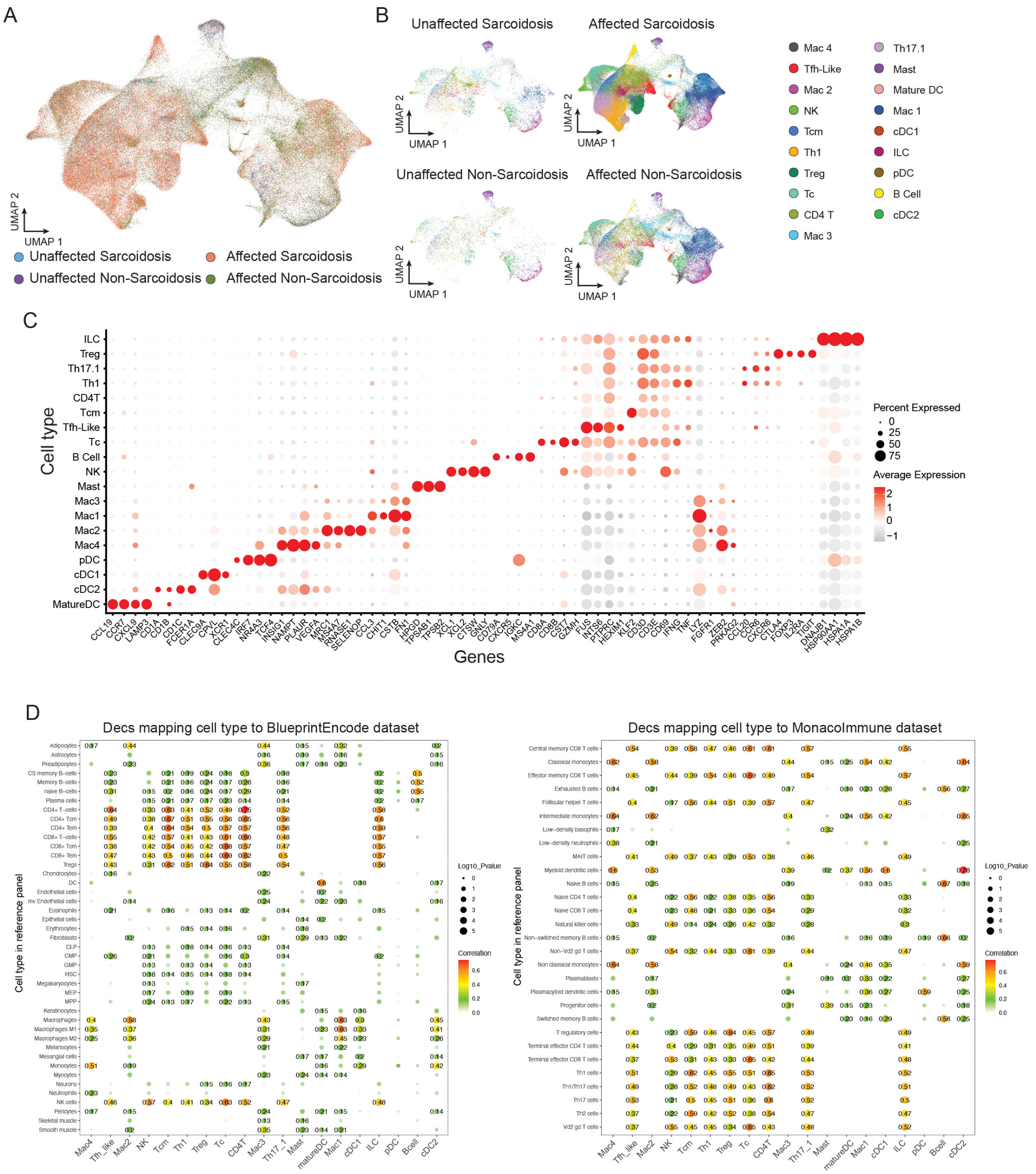

Figure S2: Immune cell landscape of skin granulomas.
(**A-B**) UMAP plot of immune subcluster in unaffected and affected skin granuloma samples demonstrating tight clustering (N=55). (**B**) Different immune cell identities are plotted for individual conditions. (**C**) Dot plot of key marker genes for each cell type. Color scale represents gene expression, and dot size represents percentage of cells expressing the marker gene. (**D**) deCS correlation plot for cell type enrichment analysis of different cell clusters. Y-axis: main cell types identified from BlueprintEncode (left) and MonacoImmune (right) databases. Color scale represents Pearson correlation coefficient, and dot sizes represent the -log10 transformed p value.

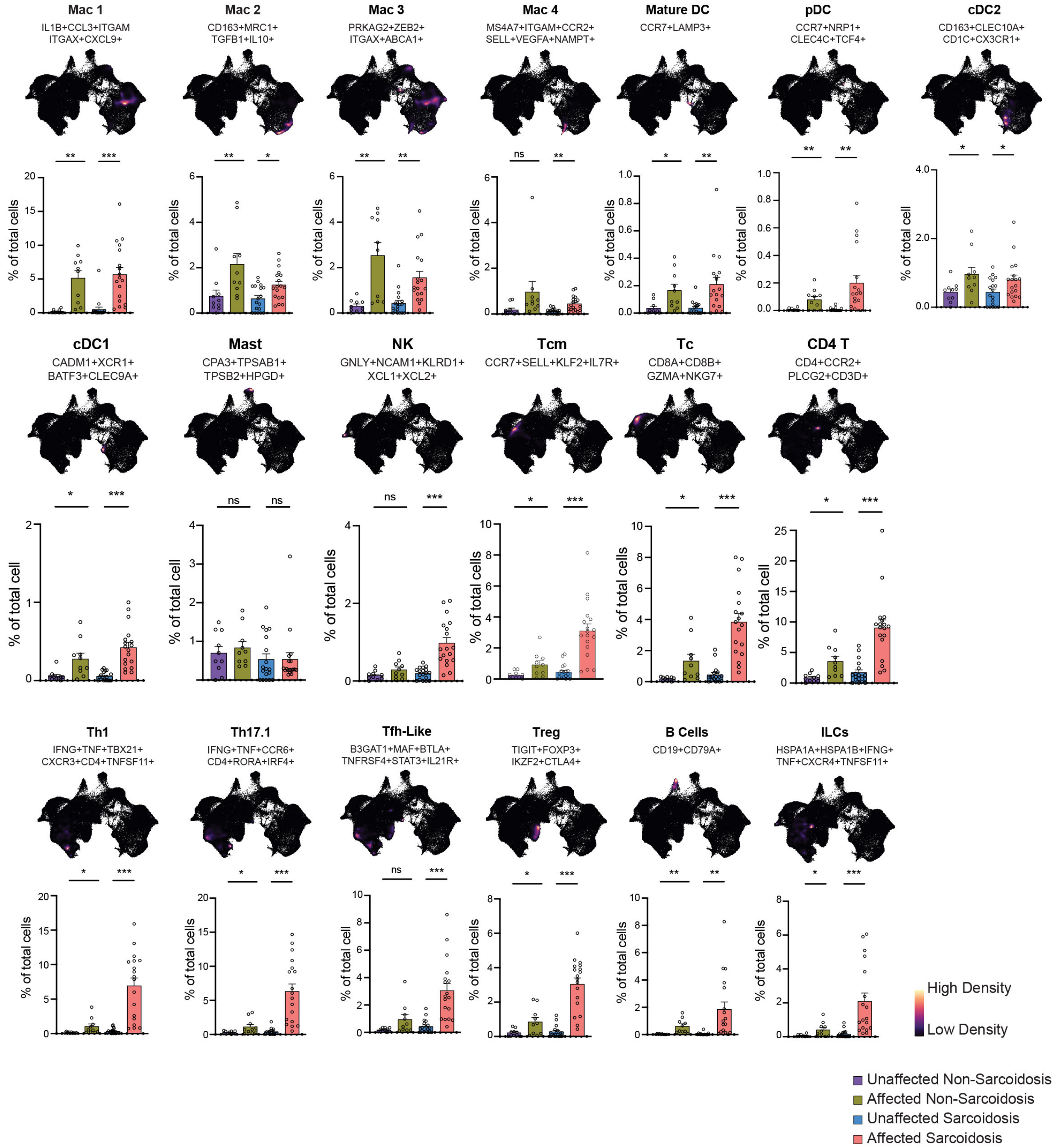

Figure S3: Individual immune cell populations in sarcoidosis and non-sarcoidosis skin granulomas.

Analysis of individual cell types with marker genes indicated below the title. Density plot demonstrates location of subgroup within UMAP. Bar plot shows relative contribution as % of total cells. DC, dendritic cell; pDC, plasmacytoid dendritic cell; cDC, classical dendritic cell; Mac, macrophage; Tc, cytotoxic T cell; Tcm, T central memory cell. Data depicted as mean ± SEM. Statistical significance was calculated using the two-tailed paired Student's t-test. *p<0.05; ** p< 0.01; p*** p<0.001.

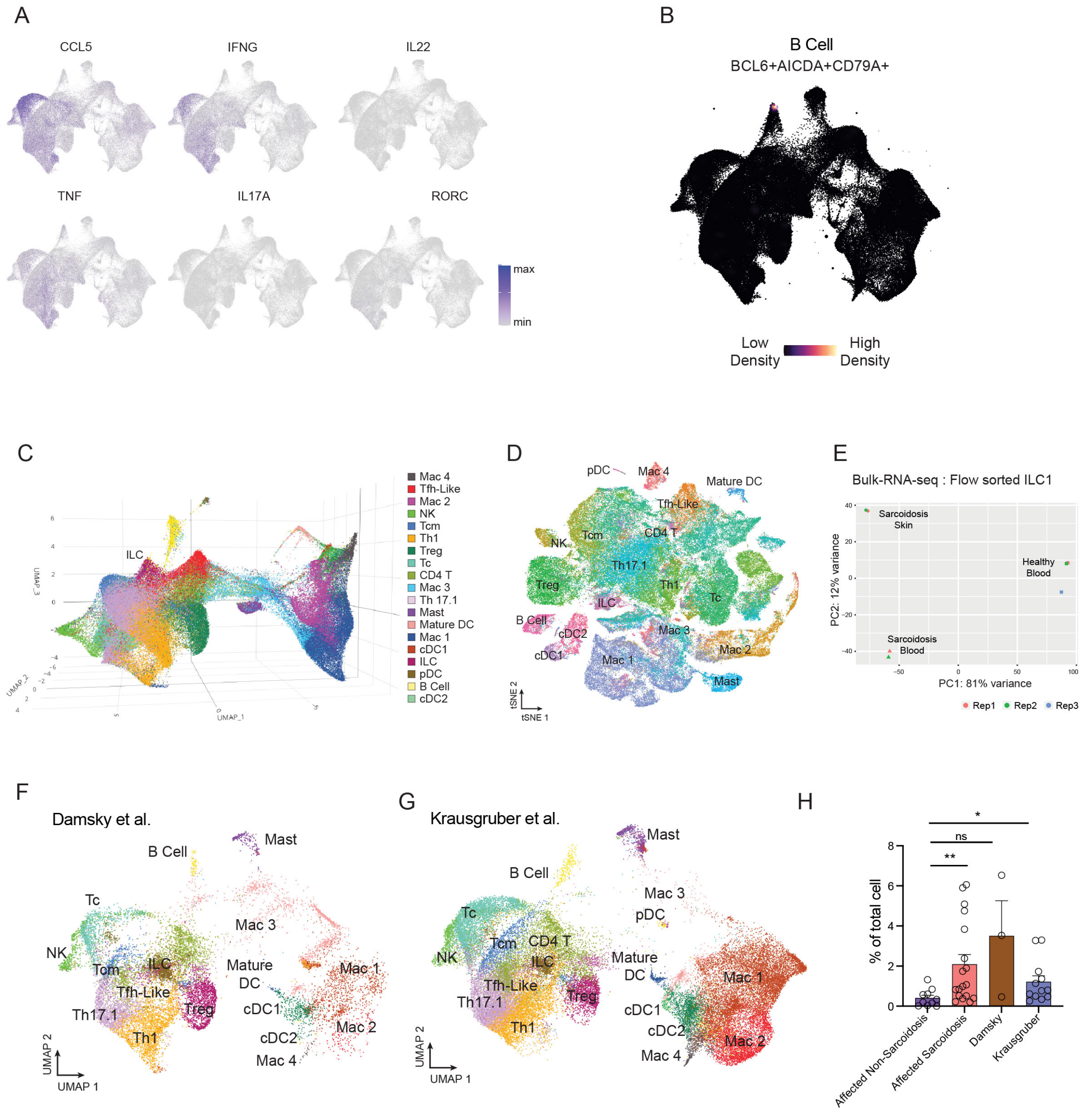

Figure S4: Sarcoidosis granulomas exhibit increased levels of ILC1s.
(**A**) Expression levels of individual cytokines within sarcoidosis UMAP. (**B**) Density plot demonstrates location of B cells expressing maturation markers *BCL6* and *AICDA* within UMAP. (**C**) 3D UMAP plot depicting subclustering of immune cells. ILCs are highlighted as a unique population. (**D**) tSNE plot depicting subclustering of immune cells. (**E**) Principle components analysis (PCA) of bulk RNA-seq samples of purified ILC1s from sarcoidosis skin, sarcoidosis blood, and healthy blood demonstrating separation between healthy and sarcoidosis samples. (**F, G**) UMAP plots of sarcoidosis affected skin from previously published datasets. (H) Bar plot shows ILC levels as % of total cells in these datasets. Statistical significance was calculated using the two-tailed unpaired Student's t-test. *p<0.05; ** p< 0.01.

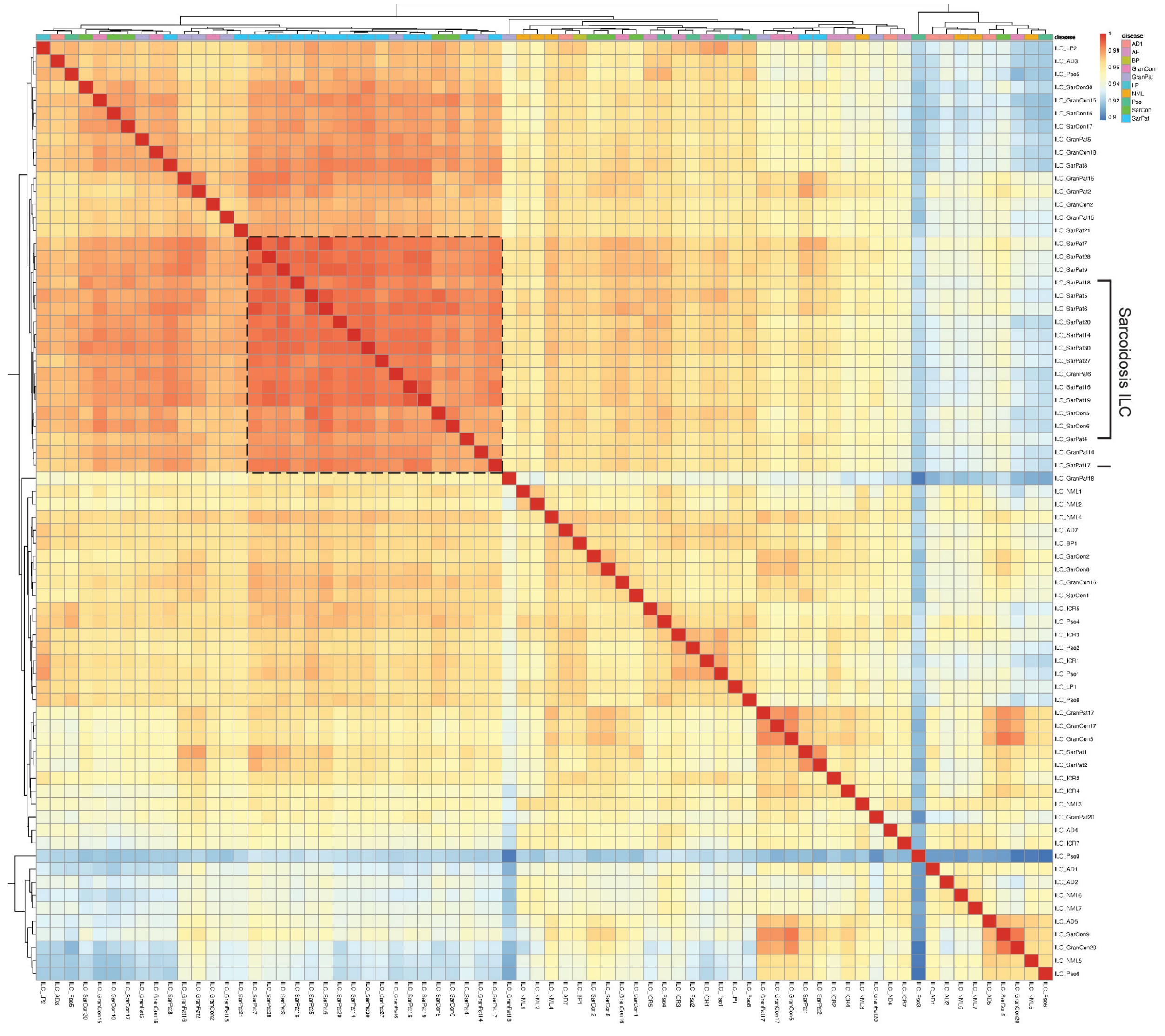

Figure S5: ILCs from sarcoidosis exhibit a unique activation pattern compared to ILCs from other dermatologic diseases. Comparison of gene activation pattern of ILCs across different dermatologic diseases and sarcoidosis (26).

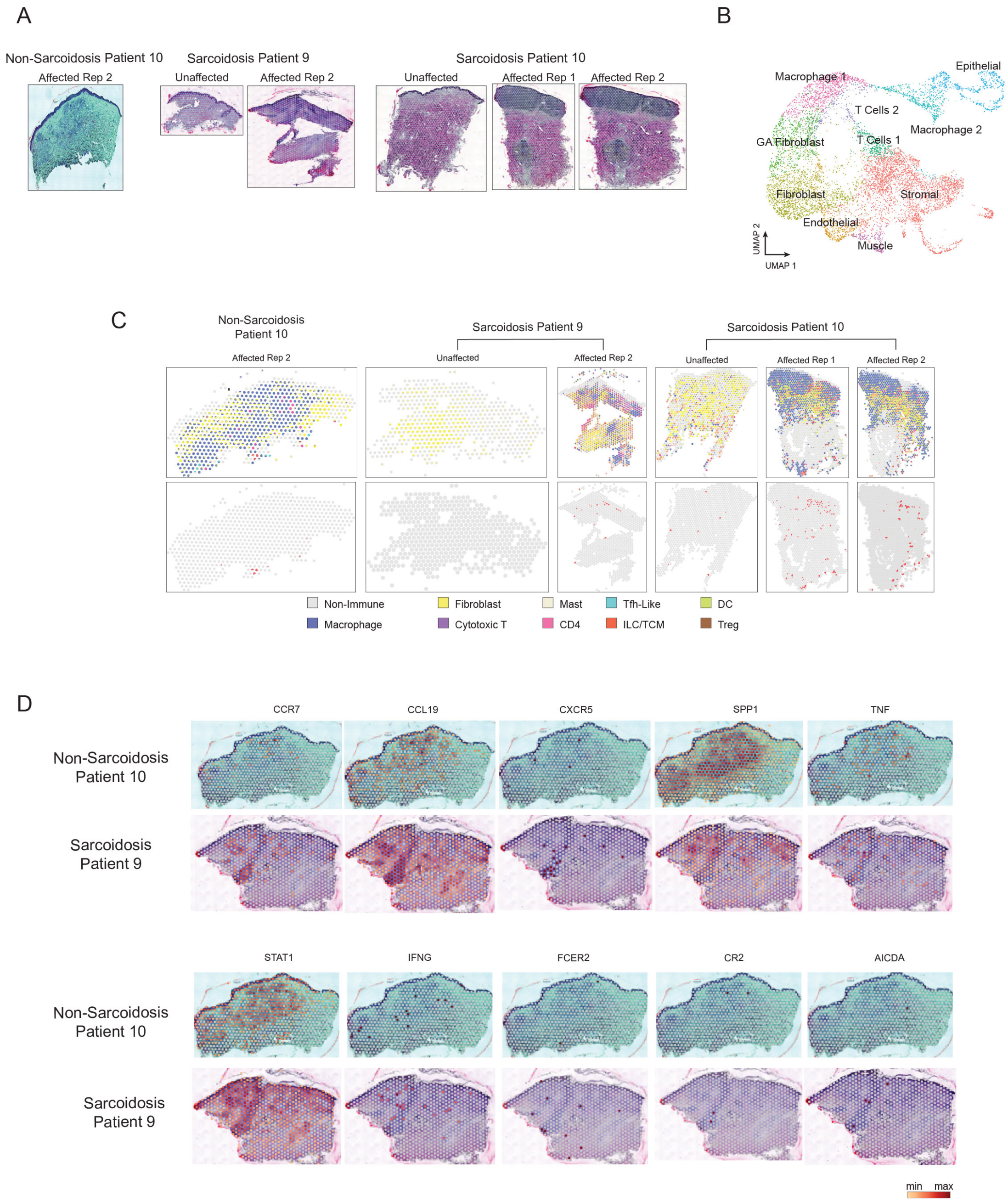

Figure S6: Spatial transcriptomics of skin granulomas.
(**A**) Representative H&E images of non-sarcoidosis and sarcoidosis patient skin granulomas. (**B**) UMAP depicting immune cell subclustering. (**C**) Deconvoluted cell type identification from spatial transcriptomics of sarcoidosis and non-sarcoidosis granuloma patients. Each spot is represented as a pie chart displaying relative cell proportions. Top panels highlight individual immune cell populations and bottom panels highlight ILCs specifically. (**D**) Expression of individual genes in sarcoidosis and non-sarcoidosis spatial transcriptomics.

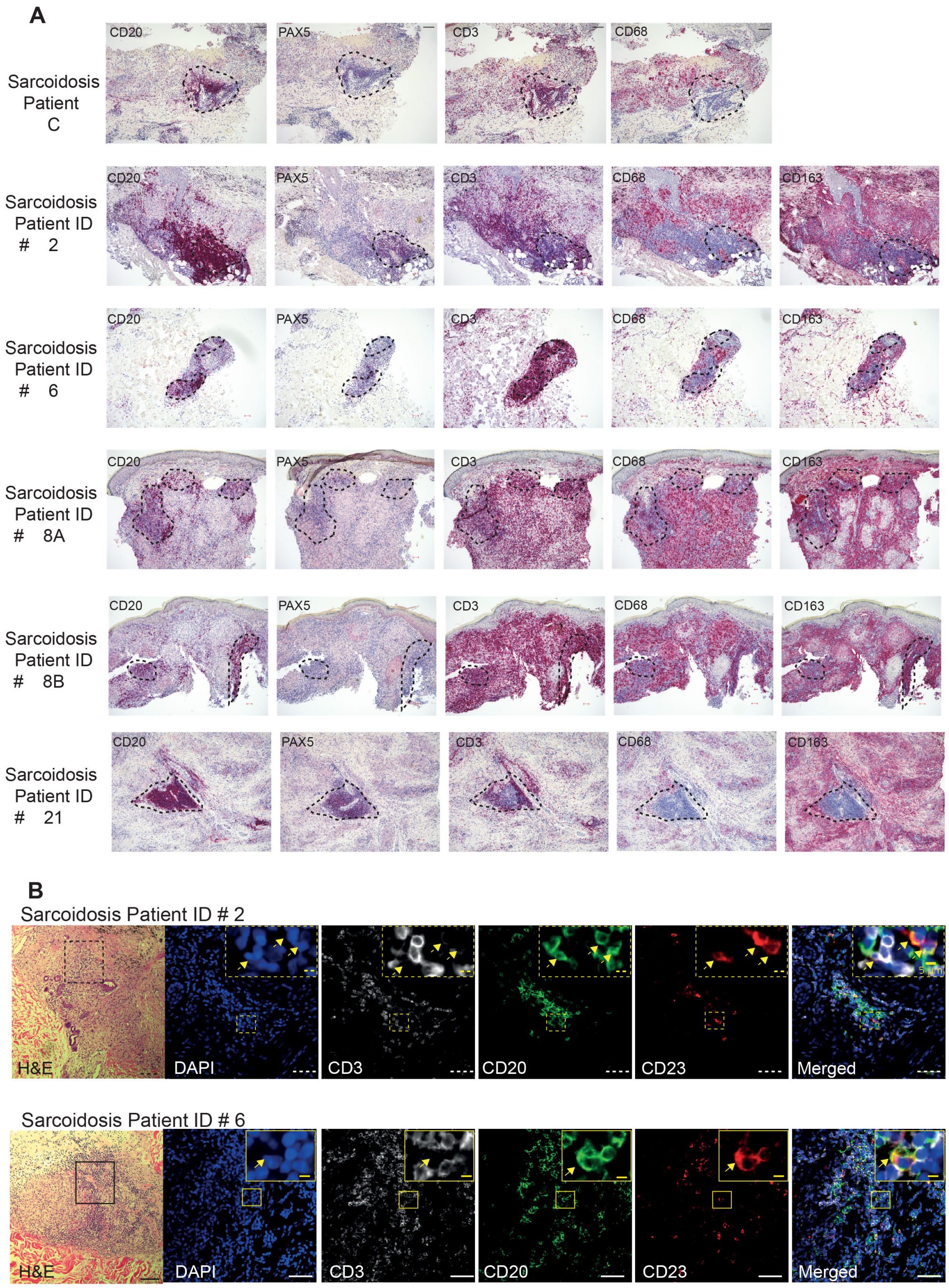

Figure S7: Sarcoidosis granulomas contain tertiary lymphoid structures.
(**A**) Immunohistochemistry depicting localization of B cells (CD20, Pax5), T cells (CD3), and macrophages (CD68, CD163) in sarcoidosis affected skin. Dotted box outlines granuloma. Scale bars: 100 µm. (**B**) Representative H&E and immunofluorescence images depicting localization of TLS (CD3–, CD20+, CD23+, yellow arrows). Scale bars: 100 µm, Scalebar in yellow inset 5 µm.

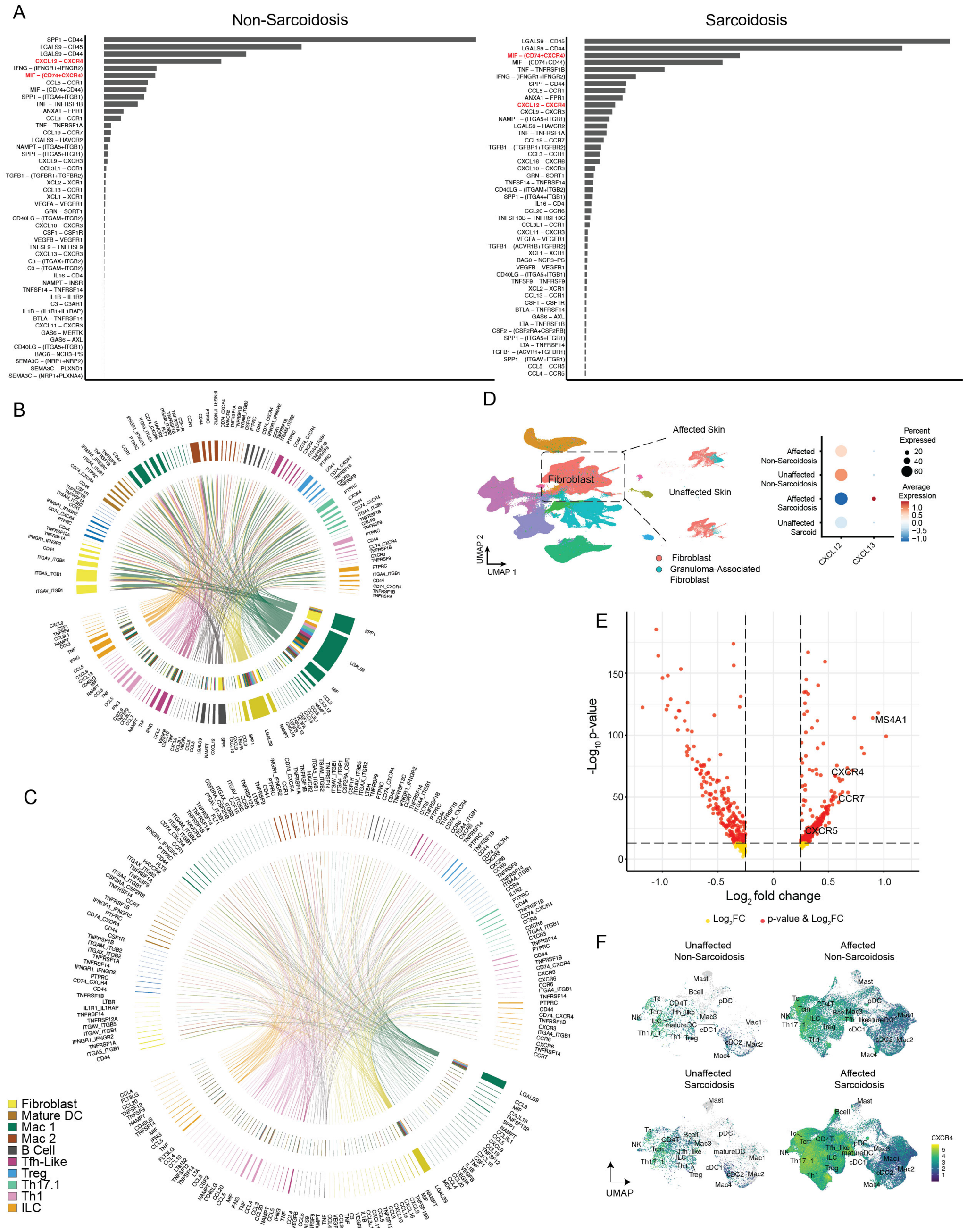

Figure S8: Sarcoidosis granulomas exhibit unique signaling changes compared to non-sarcoidosis skin granulomas.

(**A**) Global ligand-receptor analysis in non-sarcoidosis and sarcoidosis granulomas. (**B-C**) Cell-specific ligand-receptor analysis for non-sarcoidosis (B) and sarcoidosis (C) granulomas. Bottom half of the circular represents secreting cell types. Top half of the circle depicts receiving cell types. The inner bottom-half circle summarizes the receiving cell types by color. (**D**) Analysis of a specific subpopulation of fibroblasts in skin granulomas; sarcoidosis-associated populations specifically express CXCL13. (**E**) Differential gene expression of B cells in sarcoidosis affected skin highlights expression of CXCR5. (**F**) UMAP depicting expression levels of CXCR4 in sarcoidosis and non-sarcoidosis skin. Color depicts expression level.

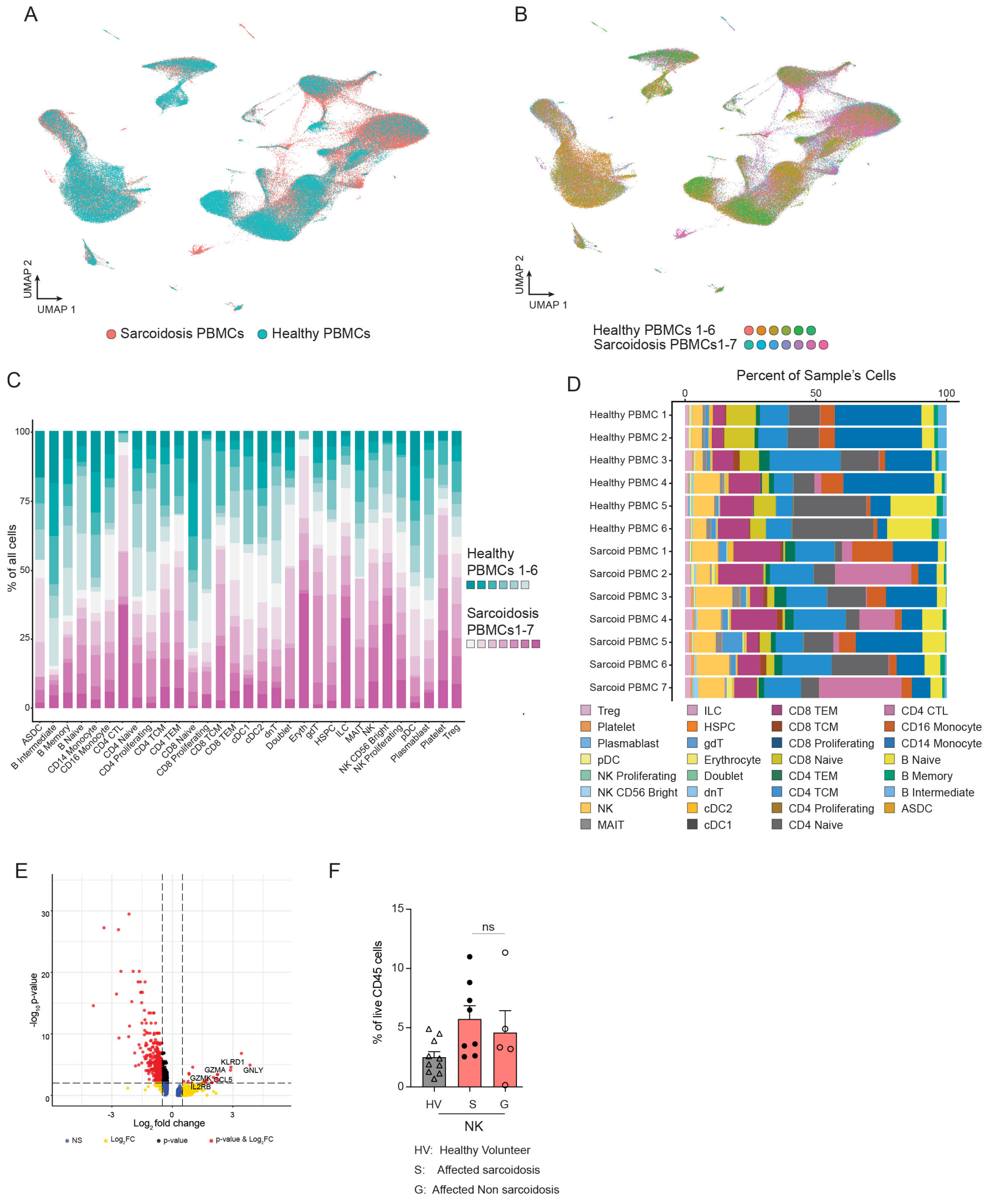

Figure S9: Sarcoidosis patients have increased circulating ILC1s.
(**A**) UMAP plots of PBMCs from healthy and sarcoidosis patients (N=6). (**B**) Cells from individual patients are annotated. (**C**) Bar plot showing relative contributions of different cell types in PBMCs. (**D**) Bar plot showing relative contribution of different cell types in individual samples. (**E**) Volcano plot of differential gene expression in ILCs from healthy and sarcoidosis PBMC samples. (**F**) NK cells levels in blood of healthy volunteers (HV), sarcoidosis patients (S) and non-sarcoidosis patients (G). Plotted as percentage of live CD45+ cells. Statistical significance was calculated using the two-tailed unpaired Student's t-test. ns, non-significant.

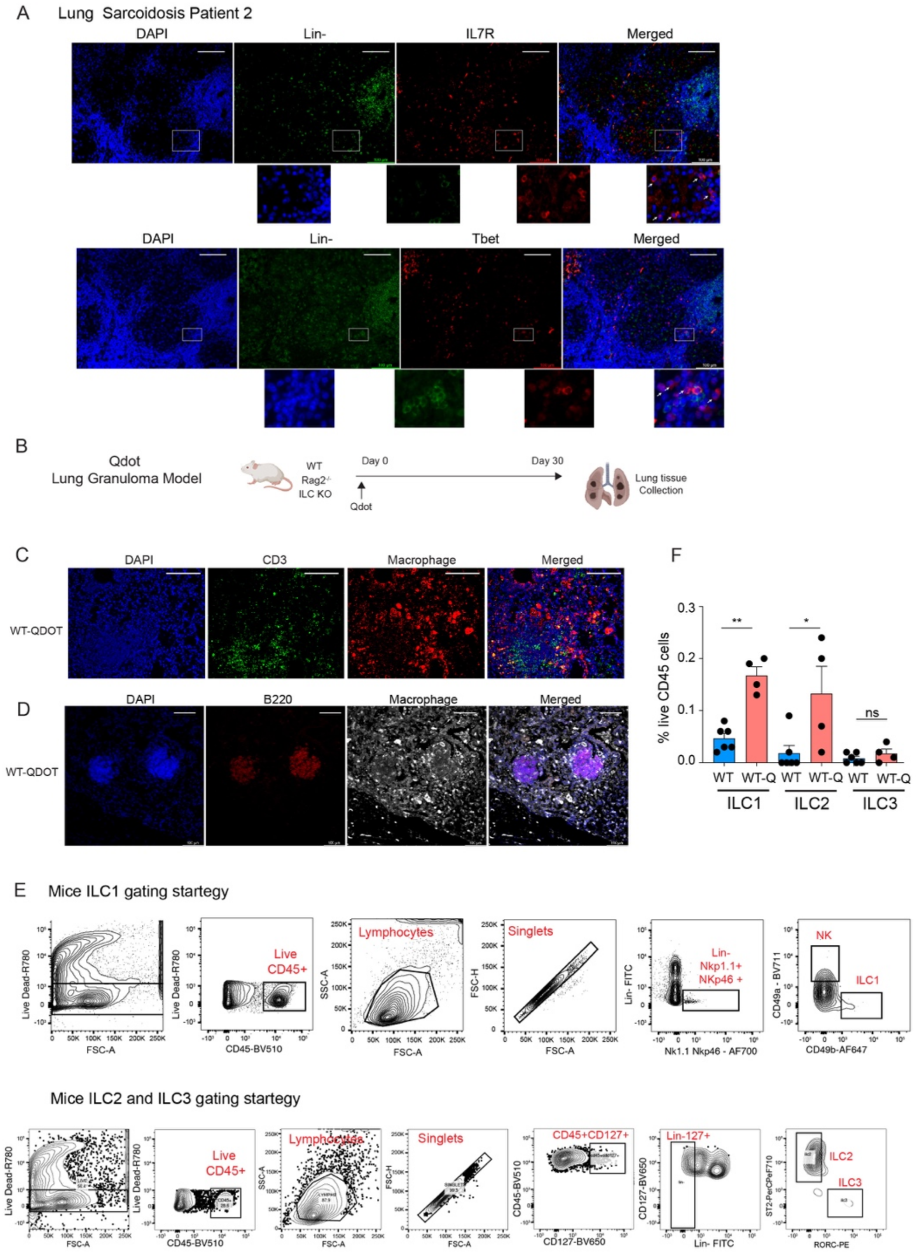

Figure S10: Lung sarcoidosis granulomas and mouse pulmonary granulomas recruit ILCs. (**A**) Representative histology demonstrating ILCs (Lin–, IL7R+, Tbet+) and recruitment of B cells (CD20+, CD23+) to human lung sarcoidosis granulomas. Lineage negative (CD3, CD16, CD19, CD20, CD56, CD68 labelled in green). Scale bars 100 µm. (**B**) Schematic of cadmium nanoparticle (Qdot) mouse model. (**C-D**) Representative immunofluorescence depicting macrophage (F4/80+), T cell (CD3+), and B cell (B220+) accumulation in lung tissue from QDOT-treated mice. The wildtype F4/80+ macrophages are also shown in Figure 6D. Scale bars: 100 µm. (**E**) Flow cytometry gating scheme for mouse ILCs and NK cells. (**F**) ILC levels in WT mice (n=6) and QDOT-treated WT mice (n=4). Plotted as percentage of live CD45+ cells. Statistical significance was calculated using the two-tailed unpaired Student's t-test. ns, non-significant. $*p<0.05$; $** p< 0.01$.

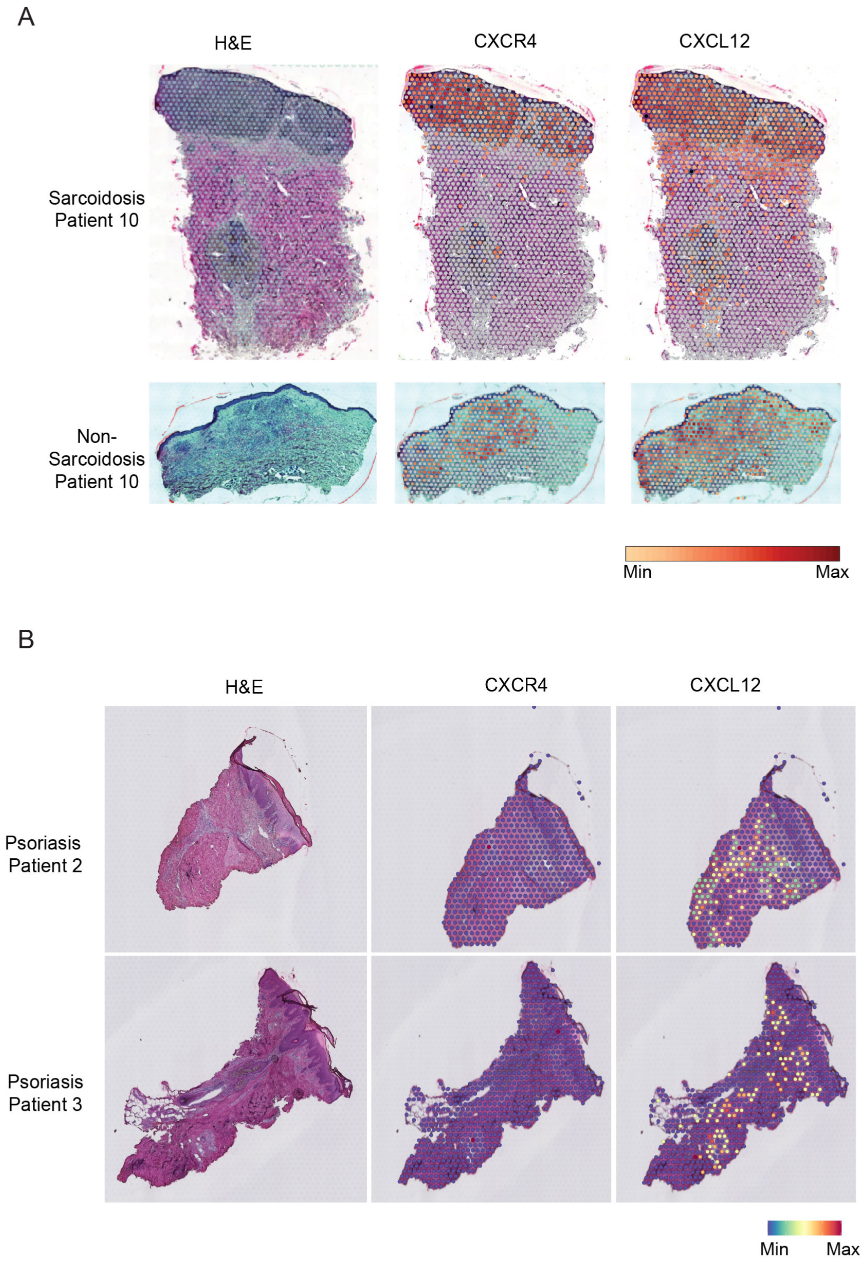

Figure S11: Sarcoidosis specifically induces CXCR4-CXCL12 signaling.
(**A, B**) Spatial transcriptomics depicting CXCR4 and CXCL12 expression in sarcoidosis-affected skin (Patient 10), non-sarcoidosis-affected skin (Patient 10), and psoriasis skin (patient 2, patient 3).
